# An Element of Determinism in a Stochastic Flagellar Motor Switch

**DOI:** 10.1371/journal.pone.0141654

**Published:** 2015-11-10

**Authors:** Li Xie, Tuba Altindal, Xiao-Lun Wu

**Affiliations:** 1 Department of Physics and Astronomy, University of Pittsburgh, Pittsburgh, PA, United States of America; 2 Department of Physics, Simon Fraser University, Burnaby, BC, Canada; The Chinese University of Hong Kong, CHINA

## Abstract

Marine bacterium *Vibrio alginolyticus* uses a single polar flagellum to navigate in an aqueous environment. Similar to *Escherichia coli* cells, the polar flagellar motor has two states; when the motor is counter-clockwise, the cell swims forward and when the motor is clockwise, the cell swims backward. *V. alginolyticus* also incorporates a direction randomization step at the start of the forward swimming interval by flicking its flagellum. To gain an understanding on how the polar flagellar motor switch is regulated, distributions of the forward Δ_*f*_ and backward Δ_*b*_ intervals are investigated herein. We found that the steady-state probability density functions, *P*(Δ_*f*_) and *P*(Δ_*b*_), of freely swimming bacteria are strongly peaked at a finite time, suggesting that the motor switch is not Poissonian. The short-time inhibition is sufficiently strong and long lasting, i.e., several hundred milliseconds for both intervals, which is readily observed and characterized. Treating motor reversal dynamics as a first-passage problem, which results from conformation fluctuations of the motor switch, we calculated *P*(Δ_*f*_) and *P*(Δ_*b*_) and found good agreement with the measurements.

## Introduction

The flagellar motor switch controlled by regulatory proteins is fundamental to bacterial chemotaxis and has broad implications for how large protein complexes work. In *Escherichia coli*, FliG, FliM, and FliN proteins form the C-ring, which is the cytoplasmic end of the rotor. About 26 FliG proteins form the upper part of a C-ring that is essential for torque generation. About 34 FliM-FliN subunits form the lower part of a C-ring that acts as a switch by interacting with CheY-P to control the direction of the motor [1]. When all subunits in the switch assume one conformation, which may be assigned as the active state, the motor rotates in clockwise (CW) direction, and when all subunits in the switch assume the other conformation, assigned as the inactive state, the motor rotates in counter-clockwise (CCW) direction. Remarkably, subunits of such a large protein complex can switch coherently and rapidly between the two conformations. The transition rate from one state, say CW to CCW or vice versa, is regulated by the response regulator CheY-P concentration, [YP], inside the bacterium [2, 3]. Observations of wild-type *E. coli* have supported the view that the flagellar motor switches stochastically in a Poissonian fashion. The Poisson behavior manifests itself in the dwell time (Δ_*CW*_ or Δ_*CCW*_) probability density functions (PDF) being exponential *P*(Δ_*s*_) = exp(−Δ_*s*_/*τ*_*s*_)/*τ*_*s*_ with the mean time *τ*_*s*_, where *s* ∈ {*CW*, *CCW*} [3–6]. Detailed biochemistry information about interacting proteins in *E. coli’*s chemotaxis network shows that [YP] is determined by external chemical signals and the state of adaption in the network [7, 8]. In order to account for the experimentally observed high sensitivity in chemosensing and fast response, cooperativity in chemoreceptors and the motor switch complex appears to be necessary [9, 10]. The classical theory taking into account these collective effects has been MWC or KNF models [11, 12]. A more general model describing protein conformation spread using Ising spins has been recently introduced [13]. This latter model allows protein conformation fluctuations to be calculated using statistical mechanics methods and is found to be in good agreement with experiments [5, 14]. When these models operate at equilibrium, i.e., constant temperature with constant transition rates between different states, both *P*(Δ_*CW*_) and *P*(Δ_*CCW*_) are sums of exponential functions and decay monotonically [15].

Herein, we report switching statistics of the polar flagellar motor of the marine bacterium *Vibrio alginolyticus* YM4 (*Pof* ^+^, *Laf* ^−^) [16]. In an aqueous environment, *V. alginolyticus* expresses a single polar flagellum that is driven by a two-state motor similar to *E. coli* [17]. When the motor turns in the CCW direction, the cell body is pushed by the flagellum, which we called forward (f) swimming, and when the motor turns in the CW direction, the cell body is pulled by the flagellum, which we called backward (b) swimming. However, unlike *E. coli* whose lateral flagellum is connected to its motor by a bent hook, the polar flagellar hook of *V. alginolyticus* is straight but bendable when a thrust is above a certain threshold [18, 19]. Consequently, at the *beginning* of each forward interval, the elastic instability of the flagellar hook induces a bent that can change the cell’s movement direction on average by Δ*θ* ≃ 90^*o*^. This conspicuous movement was termed a flick [20]. The motility pattern of *V. alginolyticus* is thus a cyclic three-step (forward-backward-flick) process; motor reversals from CCW to CW result in a kink with Δ*θ* ≃ 180°, but reversals from CW to CCW result in a broad range of angles 0 ≤ Δ*θ* ≤ 180°. Moreover, for swimming at low Reynolds numbers, the translational motion of the cell body responds to a change in the thrust force almost instantaneously [21], and the determination of a reversal event is only limited by the temporal resolution of the experimental setup. A bacterial swimming trajectory punctuated by these sequential sharp features permits us to reliably construct a time series of the flagellar motor states [19, 20]. Besides studying motor switching behaviors of free-swimming cells using video microscopy, measurements were also conducted by confining individual bacteria in an optical trap that records a cell’s position in the trap at a much higher sampling rate. Despite very different temporal resolutions of the two methods, they yield consistent results showing that the forward Δ_*f*_ and backward Δ_*b*_ dwell-time PDFs, *P*(Δ_*f*_) and *P*(Δ_*b*_), are strongly peaked at ∼270 and ∼ 370 ms, respectively. These results together suggest that the polar flagellar motor of *V. alginolyticus* is regulated in a fashion very different from *E. coli*.

## Results

### Statistical Correlations of a Polar Flagellar Motor Switch

The principal finding of our experiment is that *V. alginolyticus*’ motor reversal events are mutually exclusive, exhibiting strongly non-Poissonian fluctuations. This behavior suggests that at least one of the steps in the regulation of motor reversal is thermodynamically irreversible [15, 22]. A quick and convenient method to see this unusual behavior is by means of counting statistics commonly employed in the study of quantum particles and inter-spike intervals in neuron dynamics [23–25]. For the former case, simply counting the particle arrivals at a detector can reveal the quantum nature of the particles. If the particle arrival times are bunched together, they are bosons but if the times are anti-bunched, they are fermions. For the latter case, very useful clues about the underlying neurophysiological processes can be extracted from the observed spike train [25, 26].

**Fig 1:**
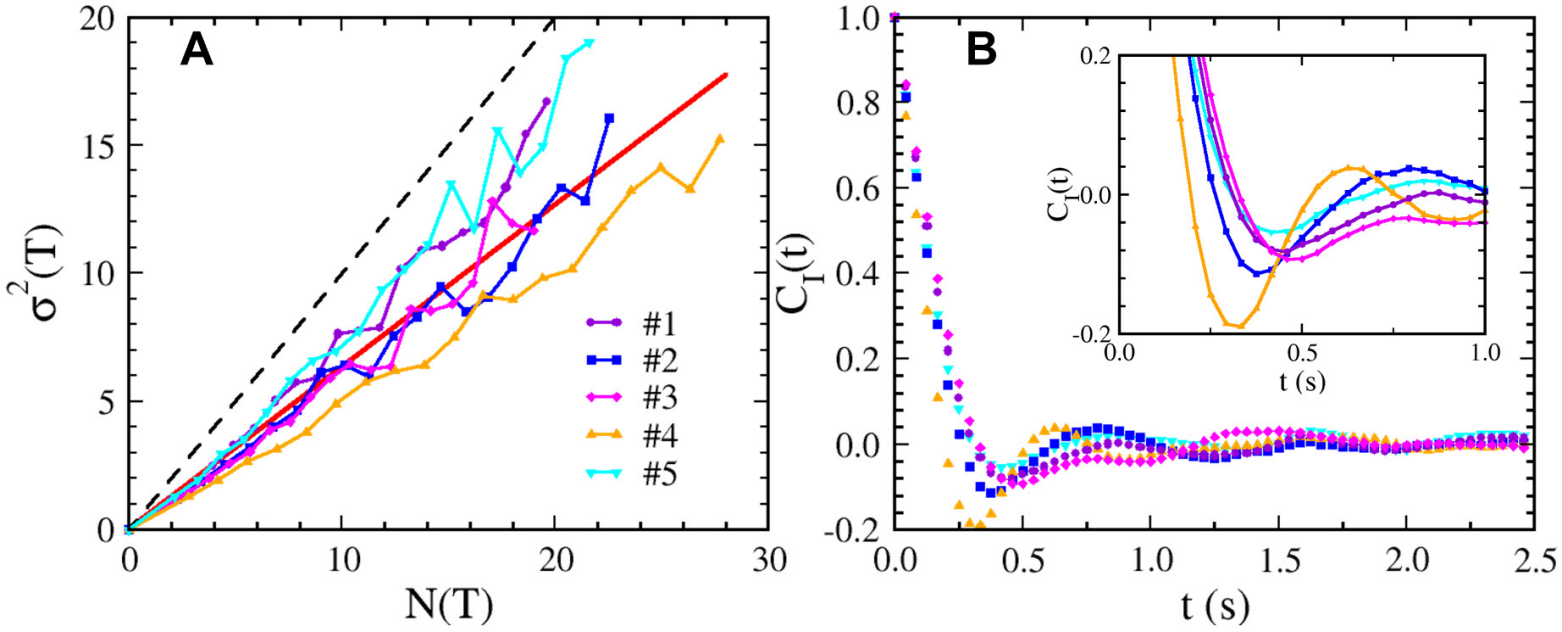
Measurements from five *V. alginolyticus* bacteria each being tracked for ten minutes. (A) *σ* ^2^ vs. *N* for five bacterial trajectories #1-#5 are displayed as indicated by the legend. The dashed black line represents *σ* ^2^/*N* = 1. Assuming that *σ* ^2^ vs. *N* is linear, a linear regression using the data from the five cells yields a straight line with a slope (or Fano factor) of 0.63, which is represented by the red line. (B) The autocorrelation functions *C*_*I*_(*t*) computed using the time series *I*(*t*′) for the five cells (see legend in (A)), where *I*(*t*′) = −1 for CW and +1 for CCW motor state (see main text). The inset displays short-time oscillations with more details.

Similarly, counting motor switching events could also shed light on the mechanism that regulates the motor direction. A useful quantity characterizing stochastic nature of the flagellar motor switch is the Fano factor, *F* = *σ* ^2^ /*N*. Here *N* is the mean count of motor reversals during time *T*, which includes both CCW to CW and CW to CCW reversals, and *σ* ^2^ is the variance. If the transition of the motor between the two states is governed by the equilibrium models such as Ref. [15], *F* ≥ 1 for *T* ≫ Δ_*f*_, Δ_*b*_. To measure *F*, five *V. alginolyticus* bacteria were randomly picked and each tracked for ∼10 minutes. Each track was then segmented into consecutive intervals of length *T*, and the average number of switches *N*(*T*) within *T*, and its variance *σ* ^2^(*T*) were calculated. Since counting is an integration process and missing an entire swimming cycle (forward+backward) is statistically unlikely based on our measured dwell-time distributions (to be discussed below), this method is not sensitive to the time resolution of the measurements. Fig 1A displays *σ* ^2^ vs. *N* for the five cells tracked. It is seen that in all cases *σ* ^2^(*T*) is smaller than *N*(*T*) or *F* < 1. The observation suggests that two consecutive motor reversal events are mutually exclusive in a fashion akin to fermions and therefore cannot be accounted for by the equilibrium model.

To characterize temporal fluctuations of the observed switching events, two types of correlation functions are computed: In the first, a binary time series *I*(*t*′) is constructed based on the state of motor rotation with *I* = +1 for CCW and *I* = −1 for CW. The autocorrelation function is defined as,

$$C_{I}\left(t\right)=\frac{\left\langle I\left(t^{\prime }\right)I\left(t^{\prime }+t\right)\right\rangle -{\left\langle I\left(t^{\prime }\right)\right\rangle }^{2}}{\left\langle I{\left(t^{\prime }\right)}^{2}\right\rangle -{\left\langle I\left(t^{\prime }\right)\right\rangle }^{2}},$$ (1)

where 〈…〉 indicates average over *t*′. If the motor switch is regulated as described by the equilibrium models, *C*_*I*_(*t*) decay monotonically with time. However, this is not what was observed in the measurement, which is displayed in Fig 1B for the five time series. We note that although *C*_*I*_(*t*) decays with time, it is non-monotonic, showing oscillations with a period slightly less than a second.

**Fig 2:**
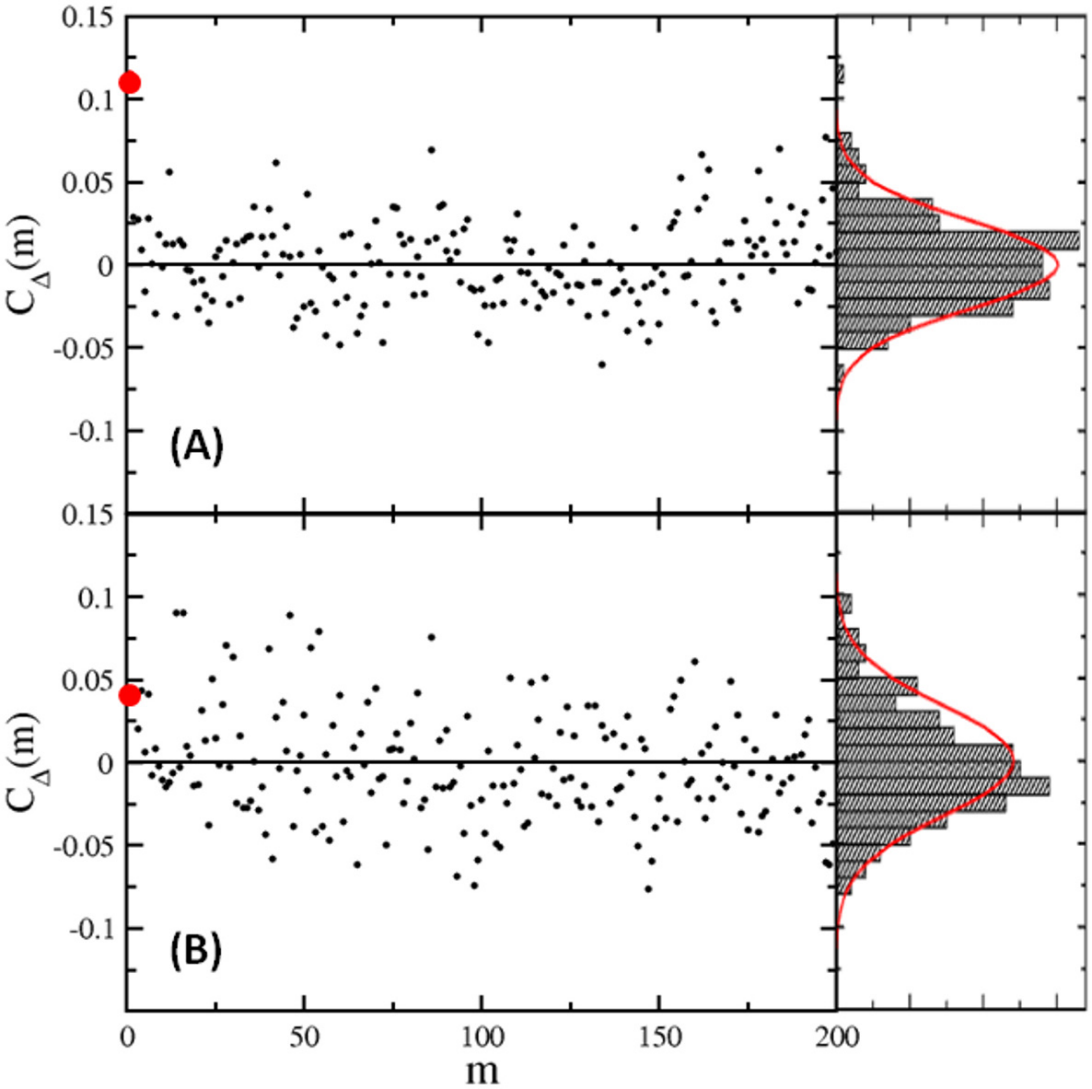
Dwell-time correlations *C*_Δ_(*m*) for cells #4 (A) and #5 (B). *C*_Δ_(*m*) vs. *m* are plotted on the left panels and the PDFs of *C*_Δ_(*m*) are plotted on the right panels. The big red dots denote *C*_Δ_(1). The red curves depict normal distributions centered at zero.

The fact that *F* < 1 and *C*_*I*_(*t*) oscillates could be a result of temporal correlations in the swimming intervals. This prompts us to examine correlations between dwell times, which is characterized by the second correlation function

$$C_{\Delta }\left(m\right)=\frac{\left\langle \Delta_{i}\Delta_{i+m}\right\rangle -{\left\langle \Delta_{i}\right\rangle }^{2}}{\left\langle \Delta_{i}^{2}\right\rangle -{\left\langle \Delta_{i}\right\rangle }^{2}},$$ (2)

where Δ_*i*_ is the waiting time between the *i* *th* and the (*i* + 1)*th* switching event, and 〈…〉 indicates average over all *i*. By this definition *C*_Δ_(0) = 1, and the next significant correlation is *C*_Δ_(1), which were found to be 0.11, 0.08, 0.09, 0.11, and 0.04, respectively for cells #1-#5. We also calculated *C*_Δ_(*m*) for *m* > 1; the data for the 4*th* and for 5*th* cell, which have the largest and the smallest *F*, are displayed respectively in Fig 2A and 2B. We note that for all fives cells, *C*_Δ_(*m*) spread randomly, and their distributions can be mimicked by the normal distributions that center at zero with the standard deviations *σ*_*C*_ varying between 0.02 to 0.04 (see Fig 2). Since *C*_Δ_(1) is overall greater than *σ*_*C*_ but much smaller than *C*_Δ_(0) = 1, the consecutive interval lengths may be correlated, but the correlation is very weak and does not extend beyond *m* = 1. Also, such correlation cannot account for the observed *F* < 1 and oscillations in *C*_*I*_(*t*). First, even though the switching sequences of cell #1 and #4 have the same *C*_Δ_(1) = 0.11, we found that *F* = 0.77 for #1 is the largest and *F* = 0.53 for #4 is the smallest among the 5 sequences studied. Second, when random shuffling is applied to the time series, the distributions of *C*_Δ_(*m*) are more-or-less unchanged, and the oscillations in *C*_*I*_(*t*) remain (see S1 Text).

### Dwell-Time Statistics Studied by Video Microscopy

#### (A) Dwell-Time PDFs Are Non-monotonic in TMN Motility Buffer

In this set of investigation we focus on the dwell-time PDFs, *P*(Δ_*f*_) and *P*(Δ_*b*_), of a large ensemble of cells, *n* ≃ 500. Because the measurements depend critically on how precisely individual motor reversal events can be determined, in Materials and Methods we provide detailed information concerning the measurement, the uncertainties, and the expected viscoelastic response times that could smear otherwise sharp transitions between the rotation states. The analysis therein demonstrated that our method can detect the motor reversal moment with adequate precision.

**Fig 3:**
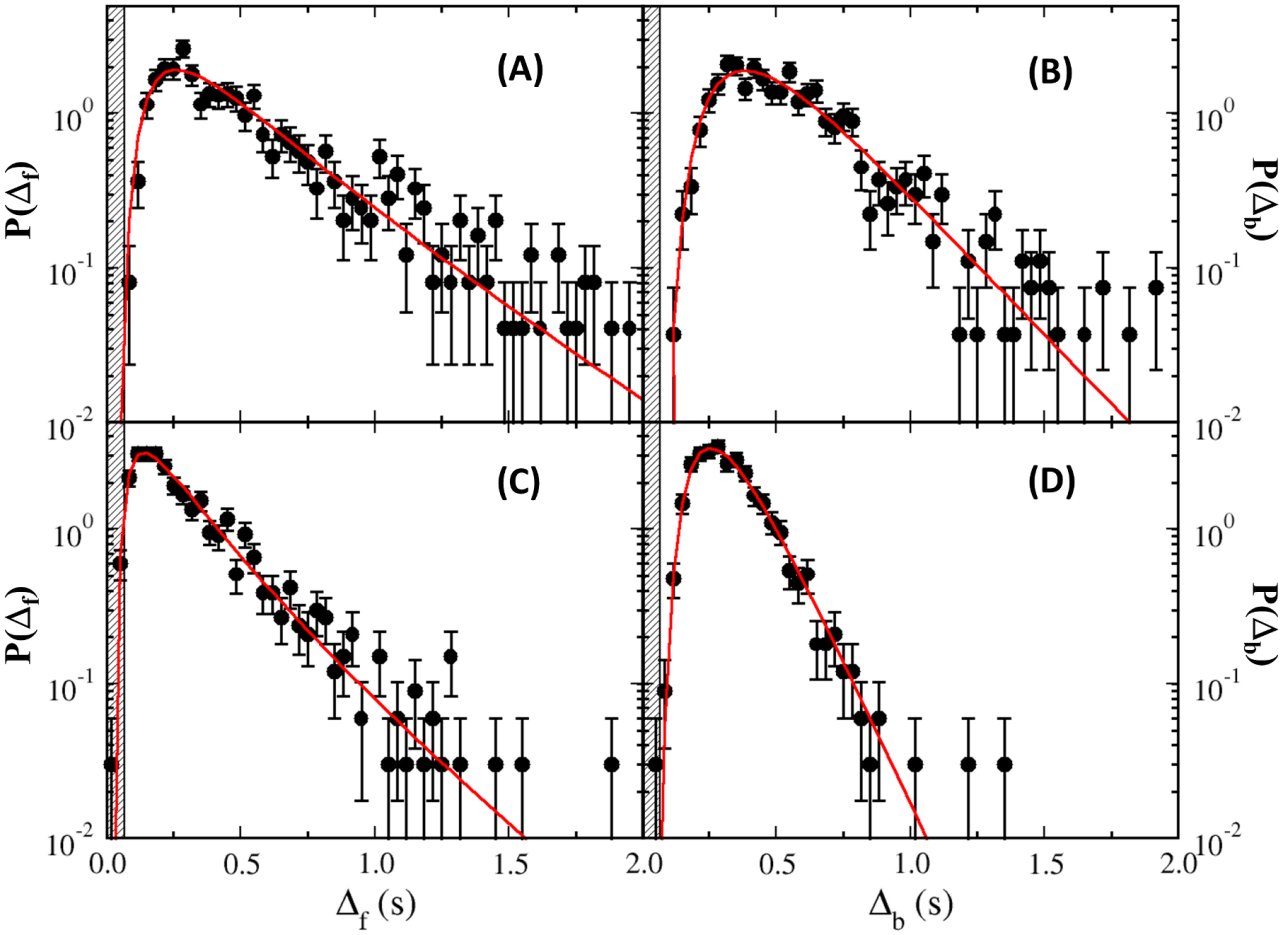
Dwell-time PDFs of YM4 in TMN (A–B) and TMN+phenol (C–D). The measurements and the fitting curves are displayed as black dots and red lines. Specifically, *P*(Δ_*f*_) and *P*(Δ_*b*_) are fitted by an inverse Gaussian distribution as described in main text. The shaded areas in these figures denote the short-time (66 ms) limitation of our measurements. Note that the time scales for the phenol data are significantly smaller than those measured without phenol.

A total of ∼500 cells’ trajectories were analyzed resulting in ∼700 − 800 individual Δ_*f*_ and Δ_*b*_. The PDFs *P*(Δ_*f*_) and *P*(Δ_*b*_) were displayed in Fig 3A and 3B, where the shaded area indicates the lower bound for the interval measurement (∼66 ms). It is evident that *P*(Δ_*f*_) and *P*(Δ_*b*_) are strongly peaked at Δ_*fmax*_ ≃ 0.27 s and Δ_*bmax*_ ≃ 0.37 s, respectively. The larger Δ_*bmax*_ suggests that spontaneous motor reversals in short times are more strongly inhibited in the CW direction than in the CCW direction. Moreover, the broad tails for both *P*(Δ_*f*_) and *P*(Δ_*b*_) make the distributions skewed towards small intervals. To a very good approximation the tail portion of the PDFs is exponential as is evident by the linear behavior seen on the semi-logarithmic plots of Fig 3A and 3B. By fitting the tails of these distributions using an exponential function, $\text{exp}(-\Delta_{s}/\tau_{s}^{\infty })$ where *s* ∈ {*f*, *b*}, we found $\tau_{f}^{\infty }=0.32$ s and $\tau_{b}^{\infty }=0.27$ s for the forward and backward intervals, respectively.

We note that the measured dwell-time PDFs for *V. alginolyticus* are significantly different from those observed in *E. coli*, which are exponentially distributed [3–6]. While there is reasonably strong evidence suggesting that *E. coli*’s flagellar motor switching is controlled by thermally activated Poisson processes [3, 5, 27], the flagellar motor switch of the marine bacterium is regulated in a decidedly different fashion. Peaking of the dwell-time PDFs seen in *V. alginolyticus* suggests that the motor switch of *V. alginolyticus* has a refractory period right after the motor has switched, i.e., within this period another motor reversal is strongly inhibited. Swimming interval times in the marine bacterium, therefore, appear to be governed by two competing processes, the short-time inhibition and long-time Poisson-like process. It comes as no surprise therefore that the oscillatory behavior seen in the correlation function *C*_*I*_(*t*) is a result of the dominant time scales, Δ_*fmax*_ and Δ_*bmax*_, in the dwell-time PDFs.

#### (B) Dwell-Time Distributions in the Presence of Chemorepellent Are Also Non-monotonic

While the above steady-state measurements are informative, revealing a significantly different switching behavior compared to *E. coli*’s flagellar motor, it is useful to see how *P*(Δ_*f*_) and *P*(Δ_*b*_) are altered when an external perturbation is applied. Most flagellar motor switches studied so far are controlled by the phosphorylated form of the regulatory protein CheY [28]. For *E. coli*, an elevated [YP] increases the switching probability from the CCW to CW state and hence the CW bias [2, 3, 5]. Regulator CheY in *V. alginolyticus* has a great deal of homology to its *E. coli* counterpart; they are 84% identical [29]. Overexpressing *cheY* or exposure to repellent phenol was shown to make the *V. alginolyticus* cell change swimming directions more frequently [29, 30]. Interestingly, this response to phenol is not adaptive in YM4 since the motor reversal rate changed by less than 10% after a 20-minute exposure [30]. This provides a convenient means to change [YP] in the cells, allowing *P*(Δ_*f*_) and *P*(Δ_*b*_) to be measured in a new steady state. The measured *P*(Δ_*f*_) and *P*(Δ_*b*_) from cells in TMN+10 mM phenol are displayed in Fig 3C and 3D). These distributions are similar to those acquired in TMN (Fig 3A and 3B) except here the time scales are significantly shortened with Δ_*fmax*_ = 0.17 s and Δ_*bmax*_ = 0.27 s. The exponential tails are characterized by $\tau_{f}^{\infty }=0.25$ s and $\tau_{b}^{\infty }=0.14$ s, respectively.

Despite very different time scales in *P*(Δ_*f*_) and *P*(Δ_*b*_) measured in the two steady states, it is remarkable that all of them can be described by the inverse Gaussian distribution. This suggests that the underlying regulation mechanisms are identical for the two steady states and for the two intervals. It is also noteworthy that incessant motor reversals at a high rate when *V. alginolyticus* is exposed to phenol is rather peculiar and is at variance with *E. coli*’s response to the same chemical. When a non-adaptive *E. coli* cell is exposed to phenol, its motor is permanently “locked” in the CW direction [31]. This extreme CW bias can be explained as a result of elevated [YP] in the cytoplasm of *E. coli* that “forces” the motor to run exclusively in the CW direction. If exposure to the repellent has the same effect of elevating [YP] in *V. alginolyticus*, for which there is little reason to believe otherwise, an inescapable conclusion is that the flagellar motor switch of *V. alginolyticus* reacts to this regulatory protein very differently from *E. coli* [29, 30, 32]. This important observation motivates a molecular toggle switch model for the polar flagellar motor to be presented in the Theoretical Modeling section.

### Optical Trapping Improves Short-Time Resolution

**Fig 4:**
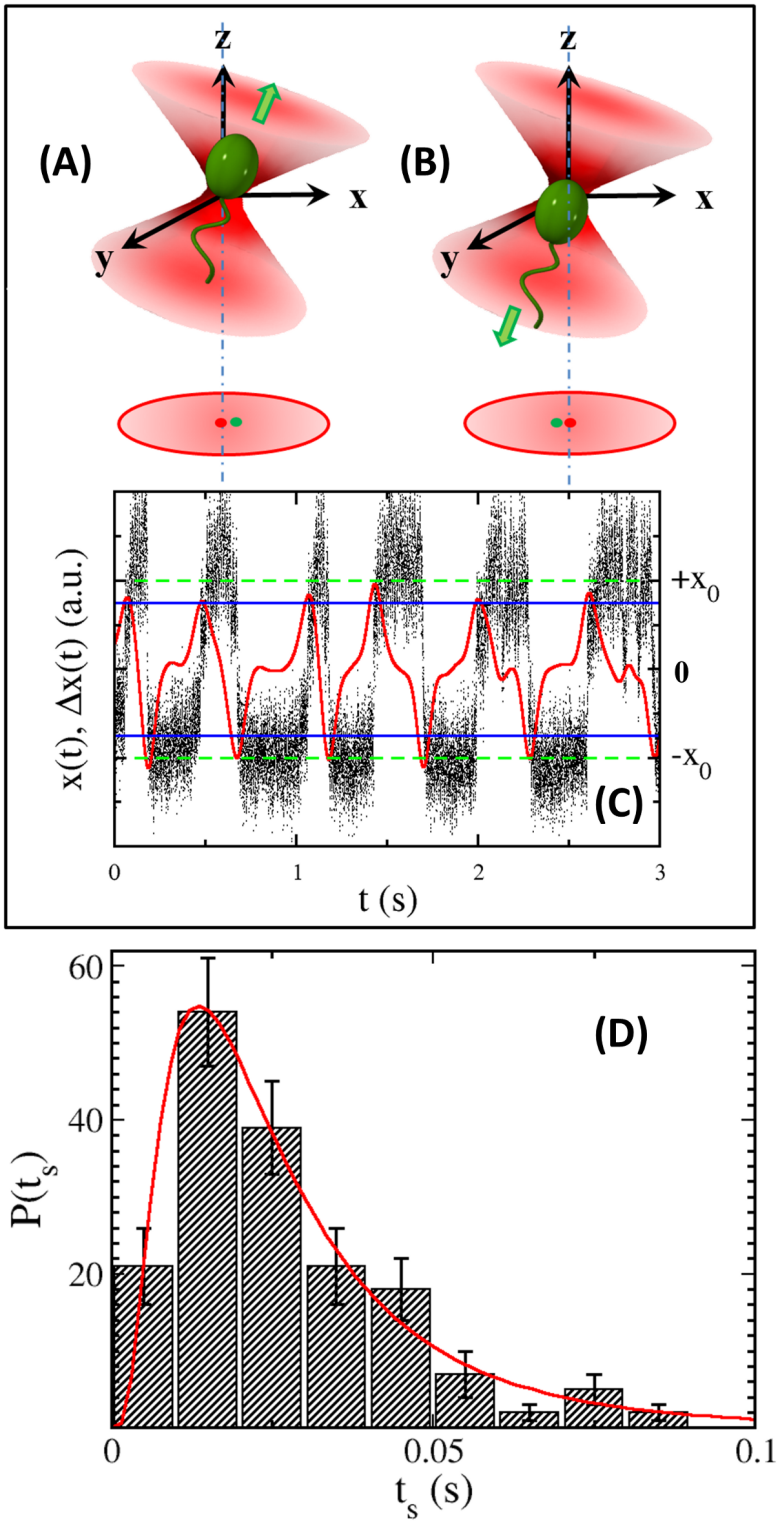
Optical trapping and swimming interval measurements. (A) depicts a forward swimming bacterium trapped at the rear end of the cell body whereas (B) depicts a backward swimming bacterium trapped at the front end of the cell body. In both (A, B), the green arrows indicate the swimming direction of the cell. This results in a shift in the center of mass of the cell in the optical trap, where the dot-dash lines and the red dots indicate the center of the optical trap. By slightly tilting the optical trap, the center of mass location of the cell body has a small projection (the green dots) along the *x*-axis of PSD. As the cell swims back and forth, its *x* position fluctuates as depicted by the small black dots in (C). There are two stable positions, +*x*_0_ and −*x*_0_, corresponding to bacterial forward and backward swimming, and they are delineated by the green dashed lines. The red curve represents convoluted time trace *Δx*(*t*), and the blue solid lines indicate the thresholds. (D) The histogram shows the transition-time distribution *P*(*t*_*s*_). The red line is the fitting to the log-normal distribution with the mean *μ*_*u*_ ≃ −3.8 and the standard deviation *σ*_*u*_ ≃ 0.72.

In this set of measurements we wish to capture fast events that might have escaped detection by video microscopy. This is achieved by capturing individual bacteria in an optical trap, where movements of the bacterium can be sampled at a rate of 10 kHz (more details in S1 Text). In the optical trap, a bacterium has two stable positions, i.e., it can be held either at the tip or at the tail of the cell body depending on its swimming direction (see Fig 4A and 4B). These two positions are readily resolved when the *z* axis is slightly tilted. In this case, the cell-body position along the *z* axis has a small projection along the *x* axis and is recorded by the position-sensitive detector (PSD) as displayed in Fig 4C [33].

**Fig 5:**
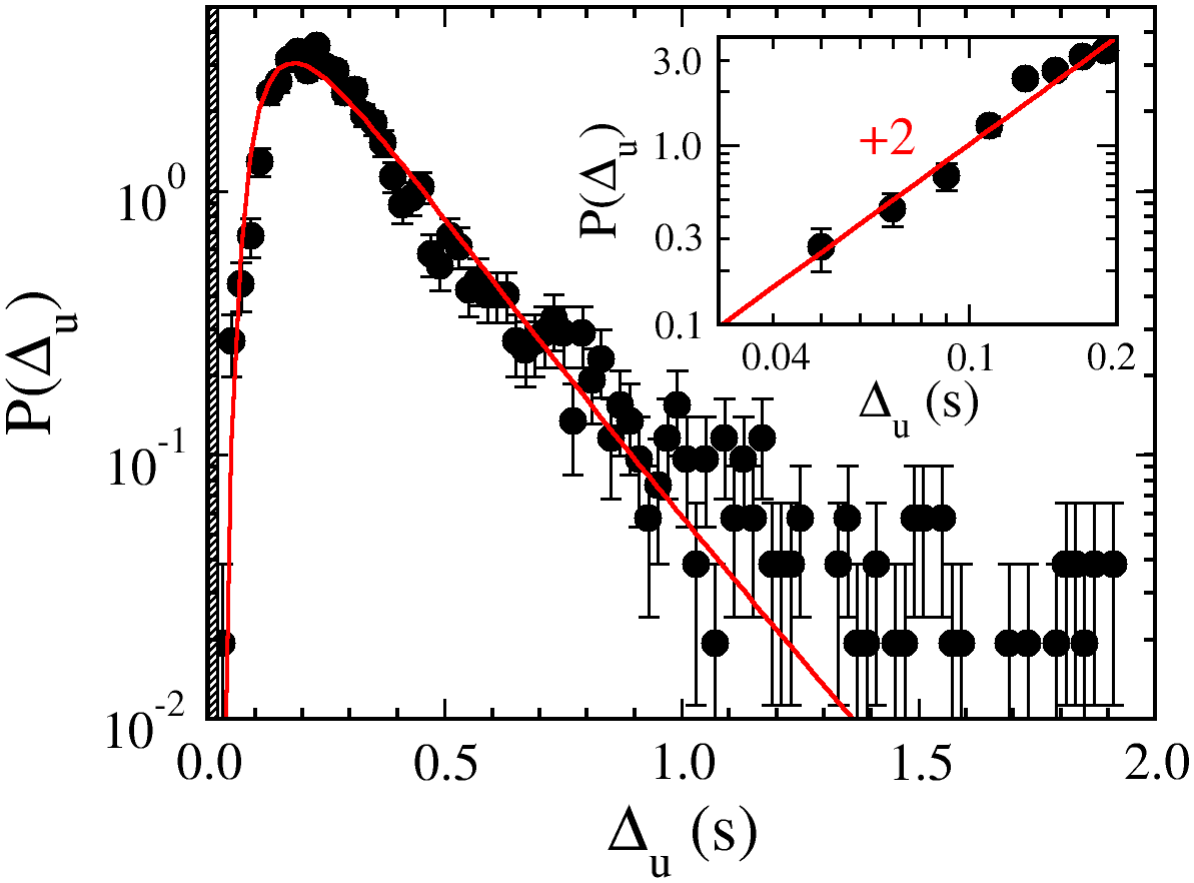
Dwell time-distribution determined by optical trapping. The undifferentiated dwell-time distribution *P*(Δ_*u*_) is represented by the black dots. The error bars indicate uncertainties in the measurement and the red line is the fit to *P*(Δ_*u*_) using the inverse Gaussian distribution. The shaded area marks the short-time detection limit $\bar{t_{s}}≃22\;ms$ discussed in the main text. The inset is a log-log plot of *P*(Δ_*u*_) vs. Δ_*u*_ for small intervals. It shows that *P*(Δ_*u*_) scales as $\Delta_{u}^{2}$.

To minimize photo-damage, cells were trapped for 3 s, resulting in 5 ∼ 10 switching events per cell. About 320 cells were analyzed with ∼3000 switching events. It is seen that the cell-body position time trace *x*(*t*) alternates between two constant levels, +*x*_0_ and −*x*_0_, with a residence time of ∼0.3 s. The upper and lower states are separated by sharp transitions that can be characterized by a transition time *t*_*s*_. We characterized *t*_*s*_ by measuring the time it takes for the signal to increase from −0.8*x*_0_ to 0.8*x*_0_ or vice versa. The PDF of *t*_*s*_ was constructed using 38 cells with a total of 172 switching events. As shown in Fig 4D, *P*(*t*_*s*_) peaks at 15ms and can be adequately fit by a log-normal distribution. The skewness of the distribution makes the mean transition time somewhat larger, $\bar{t_{s}}≃22\;\text{ms}$. Considering that the full length of *V. alginolyticus* under our culture condition is 2–3 *μ*m, and their average swimming speed is *v*_*sw*_ ≃ 55 *μ*m/s, the 22 ms transition time suggests that the bacterium moves about half of its body length in the optical trap. The mean transition time sets the temporal resolution of our trapping technique, which is about a factor of three faster than video microscopy. To detect motor reversals from a time trace, *x*(*t*) is convoluted with a smooth-differential filter *F*(*t*) = −(*t*/2*c* ^2^)exp(−*t* ^2^/2*c* ^2^), $\Delta x(t)=\int_{-\infty }^{\infty }F(t-t^{\prime })x(t^{\prime })dt^{\prime }$, and the result is displaed by the red curve in Fig 4C, where *c* = 30*ms*. The motor reversal moments are then determined whenever a peak or a valley of Δ*x*(*t*) passes the thresholds, which is set at 75% of ±*x*_0_ as indicated by the blue lines in Fig 4C. From the sequences of the motor reversal events, the dwell times Δ_*u*_ are calculated, where the subscript “*u*” indicates that the dwell time could be either Δ_*f*_ or Δ_*b*_ because it is not possible to determine the orientation of a cell in the optical trap [33]. Using this method (see more details in S1 Text), PDF of Δ_*u*_ is constructed and presented in Fig 5.

The measured *P*(Δ_*u*_) displays a fast rise for small Δ_*u*_ and an exponential-like tail for large Δ_*u*_; the deviation from a straight line in the tail is due to the fact that the interval measurement contains a mixture of Δ_*f*_ and Δ_*b*_. The measured PDF is consistent with those presented in Fig 3A and 3B, and the calculated mean dwell time ∼0.3 s also compares well with the data acquired using video microscopy. Because of the higher temporal resolution, we were able to acquire more data for small time intervals. This allowed us to examine how *P*(Δ_*u*_) behaves in the limit of small Δ_*u*_. This relationship is significant because it tells us how strongly the short time intervals are inhibited. In the inset of Fig 5, *P*(Δ_*u*_) vs. Δ_*u*_ is plotted on a log-log scale. One observes that for 50 ≤ Δ*u* ≤ 200 ms, *P*(Δ_*u*_) increases quadratically with Δ_*u*_, confirming the non-monotonic behavior seen above.

### Theoretical Analysis

#### The Dwell-Time PDFs Are Consistent with a First-Passage Time Distribution

The above experiments establish the following two important facts about the polar flagellar motor of *V. alginolyticus*: (i) Binding of CheY-P to the motor facilitates motor reversal irrespective of its current rotation state. In this sense, it behaves like a toggle switch. (ii) There exists a short refractory period within which a motor reversal is strongly inhibited. These unusual features can be significant for marine bacteria to survive in an oceanic environment and call for their quantitative understanding.

According to the molecular mechanism proposed by Paul et al., binding of CheY-P to the FliM-FliN complex induces relative movements of the subunits in the lower part of the C-ring. This causes a movement in the upper part of the C-ring that alters the stator-rotor interaction and changes the rotation direction of the motor [34]. In their view, the switch acts as a mechanical device and converts the small movements induced by CheY-P at the bottom of the C-ring into a large coherent conformational change in the upper part of the ring. Their cross-linking experiment furthermore suggests that the middle domain of the FliM proteins (FliM_M_) in a CW motor tilts relative to those in a CCW motor, causing a shift at the FliM_M_-FliM_M_ interface. Cooperativity could arise within the switch from such conformational change. Studies also showed that the FliM, FliN, and FliG proteins that form the C-ring are conserved in a wide variety of species, including *V. alginolyticus* [35, 36]. Although CheY-P affects the switching behavior of *V. alginolyticus* differently from *E. coli*, it is reasonable to assume that the switching mechanism described above is general and applicable to *V. alginolyticus*.

Below we propose a *minimal* model aimed at mimicking *P*(Δ_*f*_) and *P*(Δ_*b*_) seen in our experiment. Because the role of CheY-P on the motor is more-or-less symmetric for the two motor states [32], in the ensuing discussion it suffices to consider only one of these transitions, say from CCW to CW. We assign the conformation of the subunit to be active (inactive) when the motor is in CW (CCW) direction. Due to FliM_*M*_-FliM_*M*_ interaction, the active subunits could form a continuous domain of size *n*, and the motor switches whenever *n* exceeds a critical number *N*_*C*_.

A simple probabilistic description of finding *n* active subunits on the switch ring at time *t* is the master equation, $\frac{dp_{n}\left(t\right)}{dt}=k_{-}\left(n+1\right)p_{n+1}\left(t\right)+k_{+}\left(n-1\right)p_{n-1}\left(t\right)-\left(k_{+}\left(n\right)+k_{-}\left(n\right)\right)p_{n}\left(t\right)$, where *p*_*n*_(*t*) is such probability, *k*_+_(*n*) and *k*_−_(*n*) are rates of *n* being increased or decreased by unity. Since binding of CheY-P facilitates exiting the current motor state as deduced from our observation, we assume that *k*_+_ > *k*_−_ and they are constants. This could happen when the growth of an active domain is enhanced by binding of CheY-P. In the continuum (or large *N*_*C*_) limit, the master equation describes a biophysical process of “diffusion” with a drift

$$\frac{\partial p(x,t)}{\partial t}+V\frac{\partial p(x,t)}{\partial x}=D\frac{\partial^{2}p\left(x,t\right)}{\partial x^{2}},$$ (3)

where *x* = *n*/*N*_*C*_, $D=\left(k_{+}+k_{-}\right)/2N_{C}^{2}$, and *V* = (*k*_+_ − *k*_−_)/*N*_*C*_. The fluctuation in the domain size *n* is thus equivalent to the motion of a driven Brownian particle in a one-dimensional space, and its first-passage time distribution can be calculated by assuming that a particle is released at *x* = 0 when *t* = 0, and one measures its transmission probability at *x* = 1 as a function of *t* [37–39]. The equivalence of the two systems allows us to find the dwell-time distributions, *P*(Δ_*f*_) and *P*(Δ_*b*_), with the result

$$P(\Delta_{s})={\left(\frac{t_{Ds}}{2\pi \Delta_{s}^{3}}\right)}^{1/2}\text{exp}\left[-\frac{{\left(1-\Delta_{s}/t_{Ps}\right)}^{2}}{2(\Delta_{s}/t_{Ds})}\right],$$ (4)

where *t*_*Ps*_ = 1/*V*_*s*_ and *t*_*Ds*_ = 1/(2*D*_*s*_) with *s* ∈ {*f*, *b*}. We note that although the above derivation is for a particular scenario of the flagellar motor switch, it has a general utility for other biophysical processes that are driven by a constant “force” in a noisy environment. The thermodynamic irreversibility arises via the absorbing boundary condition imposed for solving the first-passage problem [39]. Before proceeding further, it is useful to briefly describe mathematical features of Eq 4: First, it cuts off sharply for small Δ_*s*_ and has an exponential tail for large intervals, both are qualitatively consistent with our observed dwell-time distributions. Second, Eq 4 is peaked at $\Delta_{smax}=(t_{Ps}/2)\left(\sqrt{{\left(3\gamma \right)}^{2}+4}-3\gamma \right)$ with the mean and the standard deviation given respectively by 〈Δ_*s*_〉 = *t*_*Ps*_ and *σ*_Δ_ = *γ* ^1/2^ *t*_*Ps*_, where *γ* ≡ *t*_*Ps*_/*t*_*Ds*_.

Despite its simplicity, this model describes our observations *remarkably* well as delineated by the red lines in Figs 3 and 5. Here for each PDF, *t*_*P*_ and *t*_*D*_ are the only fitting parameters, and their numerical values are listed in Table 1. We noticed that both the diffusion time *t*_*D*_ and the propagation time *t*_*P*_ depend on the state of the motor *s* ∈ {*f*, *b*} as well as the media used. Specifically, it is found that while *t*_*Pf*_ ≃ *t*_*Pb*_, there is a considerable difference between *t*_*Df*_ and *t*_*Db*_. Also, the presence of phenol in the medium significantly reduces these constants. The goodness of the fits in Fig 3 shows that *P*(Δ_*f*_) and *P*(Δ_*b*_) belong to the same family of functions.

**Table 1:** Relevant time scales of motor dynamics. The uncertainties of *t*_*P*_ and *t*_*D*_ are calculated from the estimated covariance matrix.

	*t*_*Ds*_ (s)	*t*_*Ps*_ (s)
	Forward (*s* = *f*)	
TMN medium	1.06 ± 0.06	0.50 ± 0.02
TMN+10 mM phenol	0.52 ± 0.02	0.31 ± 0.01
	Backward (*s* = *b*)	
TMN medium	2.2 ± 0.1	0.55 ± 0.01
TMN+10 mM phenol	1.79 ± 0.05	0.34 ± 0.01

## Summary

In this study we have witnessed a bacterial flagellar motor switch that operates very differently from that of *E. coli*. For *E. coli*, the regulator CheY-P behaves as a CW rotation enhancer; binding of CheY-P increases the transition rate from CCW to CW state but reduces the transition from CW to CCW state [2, 3]. For *V. alginolyticus*, on the other hand, CheY-P behaves as a switching facilitator; binding of CheY-P increases the exiting rate regardless of its current state. We posit that this type of regulation is well suited for bacteria that are capable of bidirectional swimming and chemotaxis [32].

A salient feature of *V. alginolyticus’* polar flagellar motor switch is the presence of a refractory period during which the motor reversal is strongly inhibited. This is also very different from *E. coli* for which upon switching to a new state, it can immediately switch back. Protection of a nascent state is commonly seen in digital electronics. Since high fidelity in execution of a program is so important, the “dead” time after a switch is built into logical gates of a circuit. For marine bacteria that execute the 3-step motility pattern, the “dead” time can be biologically significant. We believe that this is microorganisms’ means of combating noise, ensuring that its switching decision is not overwritten by stochastic noise in a short time. This is particularly significant in oceans where nutrients are subject to dispersion by turbulence. We note that despite stochasticity of turbulent fluid flows, dispersion of a scalar quantity in small scales are more-or-less deterministic and obeys the physical law of mixing. The existence of such mixing time allows the bacteria to develop an anticipatory response, which might explain the short-time inhibition of motor switching observed in the marine bacteria.

To illustrate the idea, we take the typical energy dissipation rate of turbulence near the surface layer of ocean to be *ϵ* ≃ 0.1 cm^2^/s^3^ and the viscosity *ν* ≃ 0.01 cm^2^/s [40]. An important spatial scale of turbulence is the Kolmogorov scale, ℓ_*η*_ = (*ν* ^3^/*ϵ*)^1/4^, which marks the termination of the inertia dominated flow and the beginning of a viscous subrange. For the given *ϵ* and *ν*, we find ℓ_*η*_ ≃ 0.06 cm. Marine bacteria live in a world in which the typical length scale they sense is less than ℓ_*η*_. Consider now a nutrient patch that is dispersed by turbulence. If for the scales ℓ < ℓ_*η*_ the nutrient is uniformly distributed, the bacteria may just give up chemotaxis because searching has no benefit. However, owing to the molecular diffusivity *D*_0_ of small nutrient molecules being typically several thousand times smaller than the kinematic viscosity *ν* of sea water, the nutrients are not distributed uniformly, but rather in patches and striations similar to the stirred milk in a coffee mug. Turbulence causes these spatial inhomogeneities to thin and eventually dissolve at a scale $\ell_{C}={\left(\nu D_{0}^{2}/\epsilon \right)}^{1/4}$, which is known as the Batchelor scale [41]. A back-of-the-envelope calculation for small amino acids, such as serine (*D*_0_ ≃ 900 *μ*m^2^/s), shows ℓ_*C*_ ≃ 17 *μ*m. Thus over a range of spatial scales ℓ_*C*_ < ℓ < ℓ_*η*_ (or 20 < ℓ < 600 *μ*m for the present case), known as the viscous-diffusion subrange, the marine bacteria can benefit from non-uniform distribution of nutrients if an appropriate chemotactic strategy is employed. We note that since ℓ_*C*_ ∝ *ϵ* ^−1/4^, the higher the turbulence intensity the smaller the dissolving scale ℓ_*C*_. Moreover, because of the small (1/4) exponent, the *ϵ* dependence is weak, and we expect that ℓ_*C*_ ≃ 20 *μ*m should not change much under different conditions. Thus, it is reasonable that for a bacterium to follow changes in a nutrient field, it has to swim the minimal distance ℓ_*C*_ because otherwise the chemical landscape is featureless. Because the typical swimming speed of a marine bacterium is *v*_*sw*_ ≃ 100*μ*m/s [42], it follows that the persistent swimming time should be ∼0.2 s. This agrees rather well with the peak positions of *P*(Δ_*f*_) and *P*(Δ_*b*_) seen in our experiment. The biological and ecological implication of the above observation is significant as about 90 percent of motile marine bacteria are polar-flagellated and possibly regulate their flagellar motors in a similar fashion [43]. This should be studied in future experiments.

## Materials and Methods

### (A) Dwell Time and Uncertainty Determination

The marine bacterium *V. alginolyticus* is a 3-step swimmer with a unique motility pattern; the recorded bacterial trajectories consist of a distinctive pattern of run, reverse, and flick, allowing identification of bacterial orientation. Even though the run (f) and reversal (b) intervals are stochastic, the cyclic 3-step pattern is distinct, facilitating tracking and identification of individual motor reversal events.

#### Tracking an Ensemble of Bacteria

**Fig 6:**
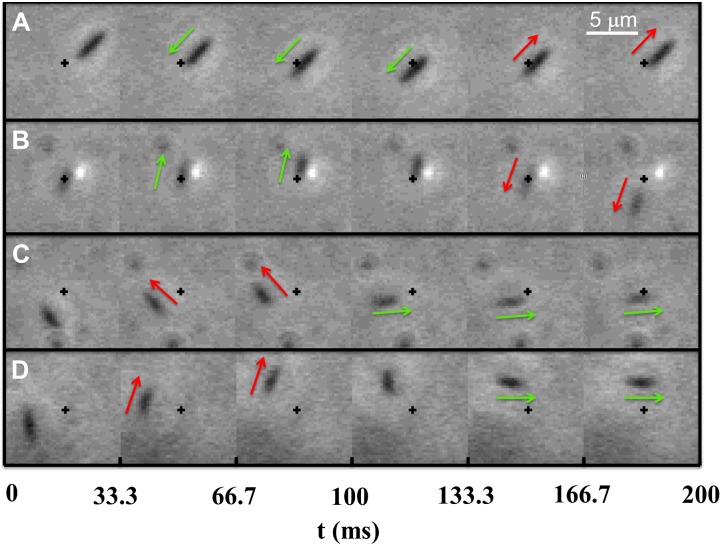
Motor reversal moments determined by video microscopy. Typical motor reversal events are displayed in (A-D), where the green and red arrows indicate the forward and backward swimming directions, and the cross is a stationary reference point. In (A, B), the transition is CCW→CW and in (C, D), the transition is CW→CCW. Note that for CW→CCW transitions, despite a large angular displacement, the translational motion of the cell body can be delayed as delineated by the 3rd and 4th frames in (D). The changes in the swimming directions along with the changes in the cell-body orientation allow the moment of the motor reversal event to be determined.

Videos of free swimming bacteria YM4 in a 10-*μ*m deep chamber were taken at the video speed of 30 fps using a 60× objective and a CCD camera (Hamamatzu, EM-CCD C9100). The image size is 512×512 pixels and each pixel measures 0.25 × 0.25 *μ*m^2^, which is close to the diffraction limit of optical microscopy. For a non-swimming *V. alginolyticus*, due to thermal diffusion, the displacement along its cell-body axis is ∼0.14 *μ*m and the angular deviation from the cell body’s semi-major axis is ∼0.11 rad in 33 ms (see S1 Text). We therefore set up a conservative criterion that during a motor reversal, if the displacement of a cell is less than 0.5 *μ*m and the cell body’s orientation changes less than 0.33 rad between the *i* *th* and the (*i* − 1)*th* frame, the motor state during the *i* *th* frame is undetermined, and the uncertainty associated with the moment of this reversal increases by ±16.7 ms. Fig 6A–6D illustrate typical examples where positions of a cell in 6 consecutive frames were displayed, showing different scenarios of CCW→CW and CW→CCW transitions (corresponding short videos can be found in S1 Video, S2 Video, S3 and S4 Videos). Denote the cell’s position in the *i* *th* frame as $\vec{x_{i}}$ and the displacement $\Delta \vec{x_{i}}={\vec{x}}_{i}-{\vec{x}}_{i-1}$. In sequence (A), since $\Delta {\vec{x}}_{4}$ and $\Delta {\vec{x}}_{5}$ are pointing in opposite directions, the cell changes its swimming direction between the 4th and the 5th frame, giving the moment of motor reversal at 133.3 ± 16.7 ms. Slower responses are occasionally observed as illustrated in Fig 6B. Here $|\Delta {\vec{x}}_{4}|$ is less than 2 pixels, while $\Delta {\vec{x}}_{3}$ and $\Delta {\vec{x}}_{5}$ are pointing in opposite directions. In this case the motor reversal moment is assigned as 116.7 ± 33 ms. In the above two cases, the transition is from CCW to CW because the displacement vectors before and after the motor reversal are anti-parallel. We noticed that the duration of the CW→CCW transition is comparable to that of CCW→CW transitions. Sequence (C) illustrates such a transition, where the cell orientation changes by ∼3*π*/4 during the 3rd and 4th frames; the moment of the flick is thus 100 ± 16.7 ms. Sequence (D) illustrates a different case where the cell body does not translate noticeably for 2 consecutive frames (66.7–133.3 ms), but its rotation is clearly discernible in the 3rd and 4th frames. The observed angle of rotation is ∼*π*/6 and is significantly greater than what would result from thermal diffusion. The transition moment in this case is assigned to be 100 ± 16.7 ms. If two adjacent transition moments are determined to be *t*_1_ ± *δ*_1_ and *t*_2_ ± *δ*_2_, the dwell time can be calculated Δ_*s*_ = *t*_2_−*t*_1_ with the uncertainty $\sigma_{s}=\sqrt{\delta_{1}^{2}+\delta_{2}^{2}}$, where *s* ∈ {*f*, *b*}. Using the above method, the smallest swimming interval that can be determined is 33 ms with an uncertainty of $\sqrt{16.7^{2}+16.7^{2}}=23.6$ ms. We found that ∼3% of *σ*_*f*_ and ∼4% of *σ*_*b*_ are larger than 66 ms (see S1 Text), which sets the limit of the resolution of swimming intervals that can be measured using the video microscopy. We note that the sequences of events recorded in Fig 6 are in good agreement with those reported in Ref. [19], where a high-speed (1000 fps) imaging method was used.

#### Tracking Individual Bacteria

For long-term observations of a single bacterium, the 10-*μ*m deep chamber was placed on a motorized stage (SD Instrument) controlled by a joystick to keep the selected cell in the field of view. The trajectory was recorded at 24 fps using a Nikon camera (Nikon D90) under a 20× objective. Due to the low magnification of the objective, a single YM4 cell can be tracked for 10 minutes (more details in S1 Text). As a trade-off, however, the resolution of motor reversal moment is reduced to ∼ 0.1 s.

### (B) Viscoelastic Response of the Propulsive Apparatus of *V. alginolyticus*

The dwell times Δ_*f*_ and Δ_*b*_ we measured are influenced by the overall response of a cell body to rotational fluctuations of the flagellar motor. It is therefore important to understand how fluctuations at the motor level affect the motion of the cell body and our measurements. Specifically, it would be interesting to know the response times of the propulsive system.

A noteworthy feature of *V. alginolyticus* is that the cell body is propelled by a single flagellum connected to a motor by a straight hook [18]. Without the need to form a bundle, the hook bending stiffness of *V. alginolyticus* is much larger than that of *E.* *coli*; e.g., *EI* for *V. alginolyticus* is ∼3.6 × 10^−26^ N⋅m^2^ whereas ∼1.6 × 10^−28^ N⋅m^2^ for *E. coli* [19, 44]. The large *EI* allows fast transmission of mechanical disturbances as the viscoelastic response time is inversely proportional to *EI*. The same can also be said about the filament as it is well known that *E. coli*’s flagellum experiences multiple morphological transformations upon motor reversals and under shear flows [45, 46]. But in *V. alginolyticus*, despite its much larger swimming speed, no morphological transformation was observed and flagellar deformation is very minute upon motor reversals [47]. Polar flagellation moreover allows the force and torque to be transmitted directly to the cell body along the body axis. This feature greatly simplifies the calculation of the response time.

Below we provide the main result of our analysis while more details can be found in S1 Text. Suppose the flagellar motor switches from CW to CCW rotation at *t* = 0, and we would like to know how long it takes for the cell body to respond to such a switch. The following sequence of events are expected: first the strains that exist on the hook and filament during the CW interval will relax, and then new strains will build up in these components when the motor rotates in the opposite direction. This process can be visualized as elastic waves propagating first along the hook, then the filament, and finally causing the cell body to move. The time scales for these events can be characterized respectively by *τ*_*h*_, *τ*_*f*_, and *τ*_*b*_, yielding the total response time *τ* = *τ*_*h*_ + *τ*_*f*_ + *τ*_*b*_. The response time for the cell body, taking into account its inertia, is very small with τ_*b*_ ∼ 10^-7^ s. The response times for the hook and the filament are also small, ∼10^−4^ s or less, when calculated using a linear elastic theory (see S1 Text). Thus, the elastic response time is expected to be τ ∼ 10^-4^ s.

The calculation above is consistent with the experimental finding of Son et al. [19] who used high-speed video imaging microscopy to investigate the mechanisms that causes *V. alginolyticus* to flick upon the motor reversal from CW to CCW (or from backward to forward swimming) reported in Ref. [20]. Recording at 1000 fps, these investigators discovered that when the flagellar motor switches from CW to CCW rotation, initially the cell body back-tracks the backward swimming path for ∼10 ms, it then sharply changes it orientation within another ∼10 ms. The short latent period, right after the motor reversal and just before flicking, is a result of unwinding and then winding of the flagellar hook; i.e., it starts from being taut, to loose, and becomes taut again. Son et al.’s measurement showed that the flexural rigidity *EI* of the hook increases by an order of magnitude when the hook is loaded as compared to when it is relaxed. The reduced *EI* makes the hook more pliable, and under compression, it buckles giving rise to a sharp turn of the cell body after a 10 ms latent period. Thus backtracking and abrupt reorientation of the cell body provides a reliable and convenient means for identifying the moment when the flagellar motor reverses, i.e., with a proper instrumentation a change in the cell body movement should be detectable at a time scale ∼ 10^-4^ s due to the viscoelastic response to a CCW→CW or CW→CCW transition. Reorientation of the cell-body, or a flick, due to the elastic instability takes a longer time with τ_*r*_ ∼ 10 – 20 ms, and conservatively we take this slow *τ*_*r*_ to be the relevant time for determining a motor reversal event using video imaging microscopy. The width of the shaded area in Fig 3 is two video frames and is about 3*τ*_*r*_.

## Supporting Information

S1 Text Additional data analysis and experimental methods. (PDF)

S1 Video A fast CCW→CW motor reversal event. This video shows a *V. alginolyticus* cell switching from forward to backward swimming when its flagellar motor switches from CCW to CW rotation, as depicted in Fig 6A.
(AVI)

S2 Video A slow CCW→CW motor reversal event. This video shows a *V. alginolyticus* cell switching from forward to backward swimming when its flagellar motor switches from CCW to CW rotation, as depicted in Fig 6B.
(AVI)

S3 Video A fast CW→CCW motor reversal event. This video shows a *V. alginolyticus* cell switching from backward to forward swimming when its flagellar motor switches from CW to CCW rotation, as depicted in Fig 6C.
(AVI)

S4 Video A slow CW→CCW motor reversal event. This video shows a *V. alginolyticus* cell switching from backward to forward swimming when its flagellar motor switches from CW to CCW rotation, as depicted in Fig 6D.
(AVI)
