# Supplementary material for: An Element of Determinism in a Stochastic Flagellar Motor Switch
Source: PLoS One. 2015 Nov 10;10(11):e0141654. doi: 10.1371/journal.pone.0141654 (PMC4640873; doi:10.1371/journal.pone.0141654)
Supplement: S1 Text — (PDF) [file pone.0141654.s001.pdf]

An Element of Determinism in a Stochastic Flagellar Motor Switch
S1 Text

Effect of Correlation Between Adjacent Intervals

When there is no correlation between switching events that are governed by the equilibrium model, the autocorrelation function $C_I(t)$ decays monotonically. However, small but discernible correlations between adjacent intervals have been observed in cells #1 and #4 with $C_\Delta(1) = 0.11$, while the standard deviation of $C_\Delta(m)$ is 0.03-0.04 for $m > 1$ (see Eq. 2 for definition of $C_\Delta(m)$). Although such correlation cannot account for $F < 1$, it can cause $C_I(t)$ to oscillate. To test this possibility, we shuffled the intervals in different ways to evaluate the effect of temporal correlation of switching events on $C_I(t)$. The time series for the #4 cell is chosen because the corresponding $C_I(t)$ has the strongest oscillation. First, to eliminate the correlation between adjacent intervals, forward and the backward intervals Δ_f and Δ_b are shuffled among themselves to generate twenty randomized binary sequences $I'(t')$. Autocorrelation functions were then computed and averaged, yielding $C'_I(t)$ that is displayed by the indigo curve in Fig. S1. As can be seen, even in the absence of adjacent-interval correlation, $C'_I(t)$ still oscillates. However, $C'_I(t)$ does deviate from $C_I(t)$ noticeably and the deviation is consistent with the result $C_\Delta(1) = 0.11$. We next shuffled time-ordered pairs (Δ_f,Δ_b) with each other to obtain another binary sequence $I''(t')$. This procedure maintains the correlation between Δ_f and Δ_b within a swimming cycle but the long-time correlation is destroyed. The average autocorrelation functions $C''_I(t)$ resulting from twenty such shuffles were shown by the brown curve. As seen $C''_I(t)$ is nearly identical to $C_I(t)$, suggesting that there is very little correlation between pairs of (Δ_f,Δ_b).

Another way to demonstrate the lack of long-time correlation is to calculate the distribution of $C_\Delta(m)$ after shuffling the forward and the backward intervals Δ_f and Δ_b among themselves, where $1 < m \leq 200$. It is evident in Fig. S2 that shuffling has little effect on the PDFs of $C_\Delta(m)$.

The above statistical analyses allow us to conclude that even though temporal correlation is discernible, these correlations exist only between adjacent intervals. The observed $F < 1$ and oscillations in $C_I(t)$ must be due to the non-monotonic distributions of Δ_f and Δ_b that show prominent peaks at finite times.

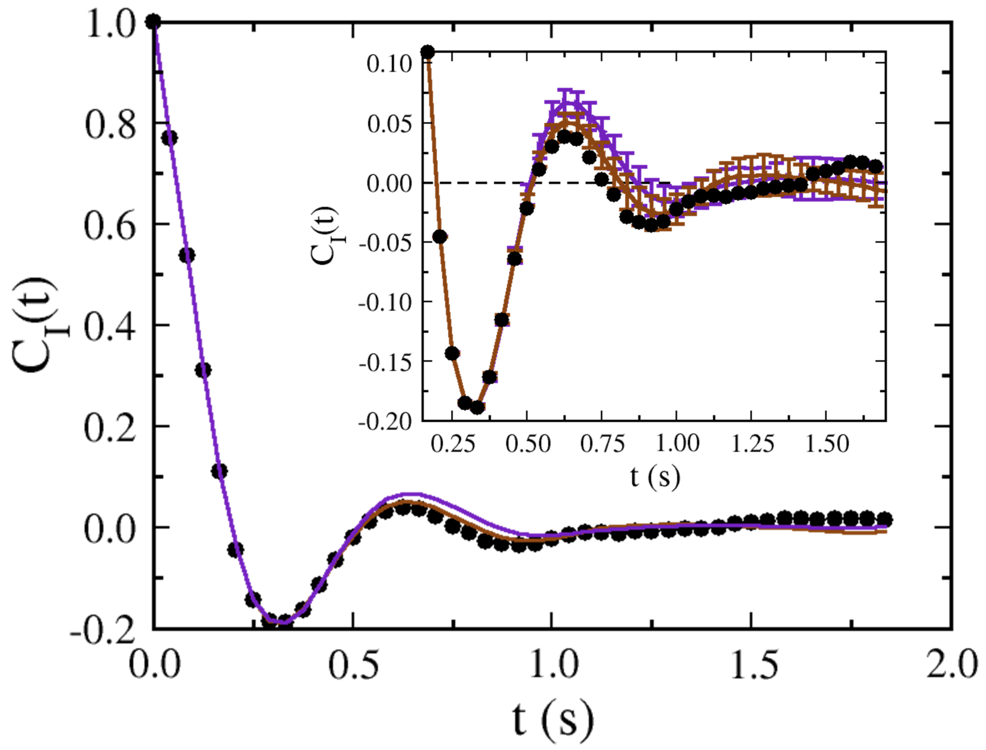

Figure S1: Autocorrelation functions of bacterial swimming intervals. $C_I(t)$ computed from the time series of the cell #4 is depicted by the black dots. The autocorrelation functions $C'_I(t)$ and $C''_I(t)$ are shown by the indigo and brown curves (see main text). To aid visualization, the region $0.2 < t < 1.5$ s was amplified in the inset. Here, the error bars for $C'_I(t)$ and $C''_I(t)$ represent the standard deviation resulting from twenty realizations of randomly shuffled time sequences.

SI Materials and Methods

Bacterial Strains and Cultures

The *V. alginolyticus* strain YM4 (Pof^+Laf^-) is a gift of Professor M. Homma [1]. The bacteria for video microscopy were grown in a minimal medium [2] (0.3 M NaC1, 10 mM KCl, 2 mM K_2HPO_4, 0.01 mM $FeSO_4$, 15 mM $(NH_4)_2SO_4$, 5 mM $MgSO_4$, 1% glycerol, and 50 mM Tris-HCl (pH 7.5)) to an optical density 0.2-0.3 at 30 °C. 1.5 mL culture was harvested and spun down at 2000×g for 3 minutes. After removing the supernatant, 1 mL TMN motility medium (50 mM Tris-HCl (pH 7.5), 5 mM $MgCl_2$, 5 mM glucose, 30 mM NaCl, and 270 mM KCl) was used to resuspend the culture followed by a 5-minute centrifuging at 500×g [1]. 300-400 μL supernatant was then carefully diluted into 2 mL TMN and shaken at 200 rpm at room temperature for at least half an hour before observation.

The bacteria for optical trapping were grown overnight in 2 mL VC medium (0.5% polypeptone, 0.5% yeast extract, 0.4% K_2HPO_4, 3% NaCl and 0.2% glucose) at 30 °C while shaken at 200 rpm. The overnight culture was diluted 1:100 into VPG (1% polypeptone, 0.4% K_2HPO_4, 3% NaCl and 0.5% glycerol) and grown for 3-4 hours at 30 °C while shaken

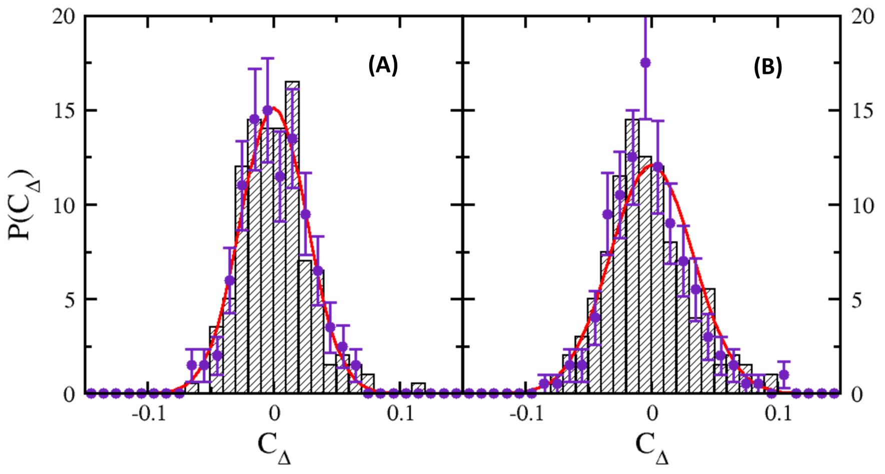

Figure S2: PDF of $C_\Delta(m)$ with and without shuffling. (A) The PDFs of $C_\Delta(m)$ calculated using the original time sequence (bars) and the shuffled sequence (indigo dots) obtained from cell #4, where $1 < m \leq 200$. The red curve is the same normal distribution shown on the right panel of Fig. 2(A). (B) The same quantities obtained using cell #5.

at 200 rpm [3]. The cells were then washed twice with TMN medium at 900×g for 2 minutes before resuspended in TMN and incubated for 8 hours before measurement.

Video Tracking of Individual Swimming Bacteria

We took videos of *V. alginolyticus* swimming using a 20× objective (Nikon, Plane Fluor 20× N.A.=0.45) in the phase contrast mode and a Nikon D90 camera at 24 fps. The observation chamber is purchased from Hawksley (Z3BC1B) and has a depth of 10 μm. The shallow depth of the chamber and the use of the low magnification allow a relatively long term recording of individual cells in the field of view. In order to follow individual *V. alginolyticus* cells for a long time, $\sim$ 10 minutes, the observation chamber was placed on a motorized stage (SD instrument, MC2000 controller, 200 Cri motorized linear stage) controlled by a joystick. The stage was moved to keep the cell inside the field of view. These videos were analyzed using the ImageJ manual tracking plug-in. One typical bacterial trajectory is displayed in Fig. S3, showing distinctively different swimming segments during forward (green) and backward (red) intervals. Due to hydrodynamic interactions with boundaries, trajectories are usually curved [4, 5]. As observed in Fig. S3, the forward swimming segment is curved in the CCW direction but the backward swimming segment is curved in the CW direction. Observations also show that the backward segments curve more strongly than the forward ones [4]. Moreover, when a cell switches from forward to backward swimming, the cell body's orientation is more or less the same. On the other hand, when a cell switches from backward to forward swimming, it usually flicks. During the flicking, the cell slows down and there are abrupt changes either in the cell body orientation, the shape, or both. Using these criteria, most motor reversal events can be determined as CCW→CW or CW→CCW without ambiguity. Those reversal events that are difficult to determine can then be identified based on the fact that the motor alternates between CCW and CW rotations. As the

trade off of the long observation time, the temporal resolution of the switching moments is not as good as when the videos are taken at 30 frames per second using a 60× objective. These long bacterial trajectories are suitable for analysis such as estimating the Fano factor and temporal correlation where uncertainties are averaged out, and they were not used for calculating $P(\Delta_f)$ and $P(\Delta_b)$.

An often used method in *E. coli* studies is the rotation assay, which relies on tethering the cell body or a flagellum to a surface. Although convenient, the method can skew motor switching behavior as recent studies indicated that switching statistics are influenced by the load [6, 7]. The use of freely swimming bacteria in this study, although tedious, is free of these complications, and the measured switching statistics directly reflect the unperturbed physiological state of the bacteria.

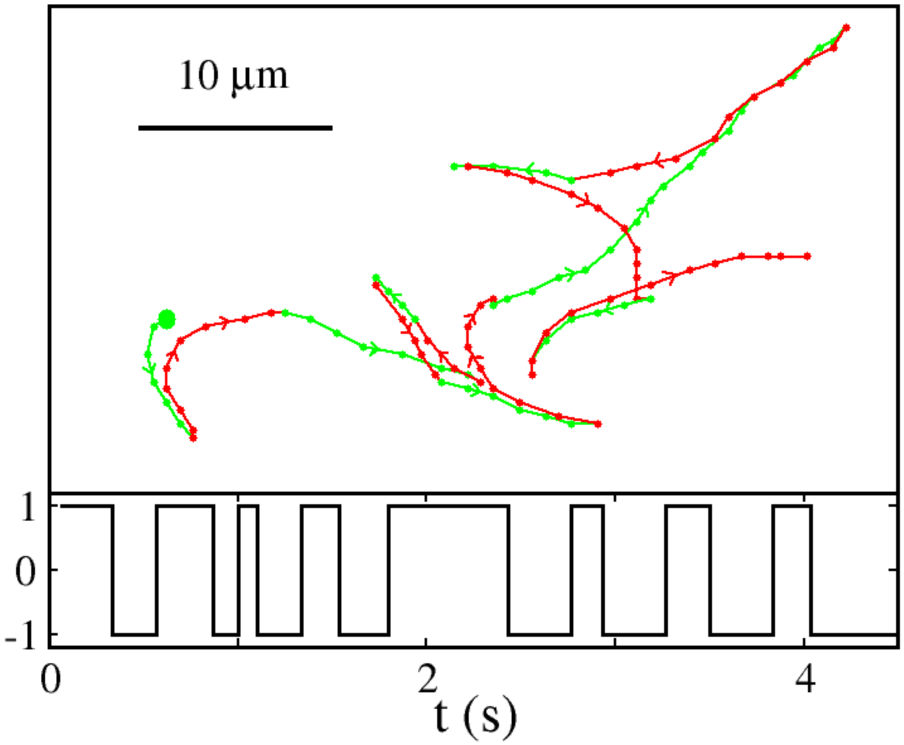

Figure S3: A typical bacterial trajectory and its binary presentation $I(t')$. The green and red lines denote the forward and the backward swimming segments, respectively. The large green dot indicates the starting point of the trajectory at $t = 0$ and the small dots are the positions of the bacterium at an equal time interval $\Delta t = 0.042\,\text{s}$. The arrows indicate the swimming direction. To aid visualization, some dots are shifted slightly to avoid overlapping. On the bottom of the figure the bacterial trajectory is binarized according to $I(t') = +1$ if the cell swims forward and $I(t') = -1$ if it swims backward.

Recording Motor Switching Events of Individual Bacteria Using Optical Trapping

A home-built optical trap was used to detect motor reversals of individual cells as described in Ref. [8]; here only the relevant aspects are delineated. Using radiation pressure from a tightly focused laser beam ($\lambda = 1054\,\text{nm}$, $\sim 50\,\text{mW}$), the elliptically shaped bacterium is trapped along the optical axis as shown in Fig. 4(A, B). The trapped bacterium has a limited range of movement ($\sim 1\ \mu$m) along the optical axis, but its rotational degree of freedom about this axis is unrestricted. Because the flagellum and the cell-body axis is rarely perfectly aligned, a swimming bacterium wobbles in the optical trap and this small irregular motion can be recorded using a two-dimensional position sensitive detector (PSD), resulting in a time series $(x(t),\, y(t))$ [9]. In our previous work, we demonstrated that by a simple Fourier transformation of $(x(t),\, y(t))$, the rotation frequencies of the cell body, the flagellum, and the moments of motor reversals can be determined [9, 8]. The same quantities may also be measured by a recently developed double optical tweezers with improved performance [10, 11].

For the present work, a similar but simpler approach is taken to identify the motor reversals. Specifically we found that by tilting the optical (z) axis of the trap, the bacterial motion along the z axis has a projection on the x axis and can be recorded by the PSD at a sampling rate of 10 kHz. In the experiment, each cell is trapped for several seconds to obtain the time trace $x(t)$. To avoid potential artifacts, as a result of photodamage to the cells, only the first 3 s of data is processed. A typical time trace is displayed in Fig. S4 (also Fig. 4(C) in the main text). The histogram constructed from $x(t)$ can be fitted to a sum of two Gaussian functions as delineated in the right panel of Fig. S4. The centers of these two Guassians, $+x_0$ and $-x_0$, correspond to the two stable positions of the bacterial cell body in the optical trap. Since the standard deviation of the Gaussian functions are about $x_0/3$, $+x_0$ and $-x_0$ are well separated despite the noise in the measurements. This allows individual motor reversals to be determined by a computer with little ambiguity.

To begin with, we measured $P(t_s)$, the PDF of ~ 180 transition times between $+x_0$ and $-x_0$. As seen in Fig. 4(D), $P(t_s)$ is peaked at $15\,\text{ms}$ and has a broad tail, yielding the mean switching time $\bar{t}_s \simeq 22\,\text{ms}$. As discussed in the main text, this time corresponds to a bacterium moving $\sim$1 μm at the swimming speed of $v_{sm} \simeq 55\,\mu$m in the trap and can be considered as the temporal resolution of the technique. Next we determined dwell times Δ_u from a time trace using a Matlab code. First, the transitions between $+x_0$ and $-x_0$ are accentuated by convoluting $x(t)$ with a smooth-derivative filter $F(t) = -\frac{t}{2c^2}\exp\left(-t^2/2c^2\right)$. Here c sets the time scale by which the raw data is smoothed and the time derivative is taken. Also, $F(t)$ is properly normalized so that when convoluted with the Heaviside function $H(t)$ it yields $\int_{-\infty}^{\infty} H(t-t')F(t')dt' = 1/2$ for $t = 0$.

In Fig. S4, the convoluted data $\Delta x(t) = \int_{-\infty}^{\infty} x(t-t')F(t')dt'$ is displayed by the red curve, where $c = 30\,\text{ms}$. The prominent maxima and minima of the curve clearly mark the moments when the motor reverses. Second, since there are also secondary maxima and minima in $\Delta x(t)$, their selection or discrimination must be decided. This is carried out by setting the thresholds $x_{th} = \pm k x_0$ with $0 < k < 1$. If a maximum or minimum of $\Delta x(t)$ exceeds the threshold, the corresponding fluctuation in $x(t)$ is counted as a switching event. Otherwise it is discriminated as noise or an incomplete switch. The blue lines in Fig. S4

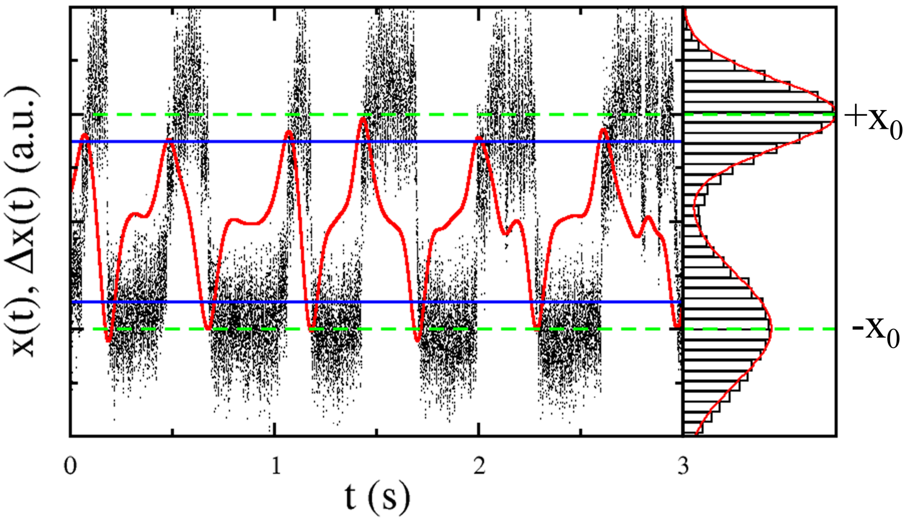

Figure S4: Determination of motor reversal moments from the time trace $x(t)$. The black dots, $x(t)$, are the x-projection of the position of a bacterium in the optical trap at every 0.1 ms. The forward and backward swimmings of the bacterium result in two stable positions of the cell body in the trap. Since the optical axis of the trap is slightly tilted, $x(t)$ fluctuates around two constant values, $+x_0$ and $-x_0$, which are marked by the green dashed lines. x_0 can be determined precisely by fitting the histogram of $x(t)$ to a sum of two Gaussian functions as delineated by the red curve in the right panel next to the time trace. To determine the moments when the motor changes its rotation state, $x(t)$ is convoluted with $F(t)$, resulting in $\Delta x(t)$, which is displayed by the red curve superimposed over the time trace. As can be seen, the major maxima and mimima match very well with the transitions of $x(t)$, and they exceed the threshold values, $x_{th} = \pm k x_0$, marked by the blue lines.

mark x_{th} with $k = 75\%$, and they clearly discriminate the prominent maxima and minima in $\Delta x(t)$ from the secondary ones. In general, the larger the k (or x_{th}) value, the more the secondary maxima and minima are discriminated.

We also investigated systematically how varying c and k affects the measured dwell-time distribution (or histogram) in the optical trap, and the result is shown Fig. S5. The panels on the top and bottom rows are obtained using $k = 75\%$ and $k = 50\%$, respectively. The three columns, from left to right, correspond to $c = 30$, 20, and 10 ms, respectively. As can be seen, reducing c or k has the same effect of introducing more short intervals into the histogram. Importantly, however, all the histograms remain non-monotonic with a prominent peak at $\Delta_u \simeq 0.2 - 0.3$ s. Taking into consideration the temporal resolution of this technique, which is $\bar{t}_s \simeq 22\,\text{ms}$, c should be comparable, or slightly greater than $\bar{t}_s$. As seen in Figs. S5(A, D), when $c = 30$ ms, both histograms obtained using $k = 75\%$ and $k = 50\%$ are not significantly different from each other, or for that matter different from the one measured using the video imaging technique.

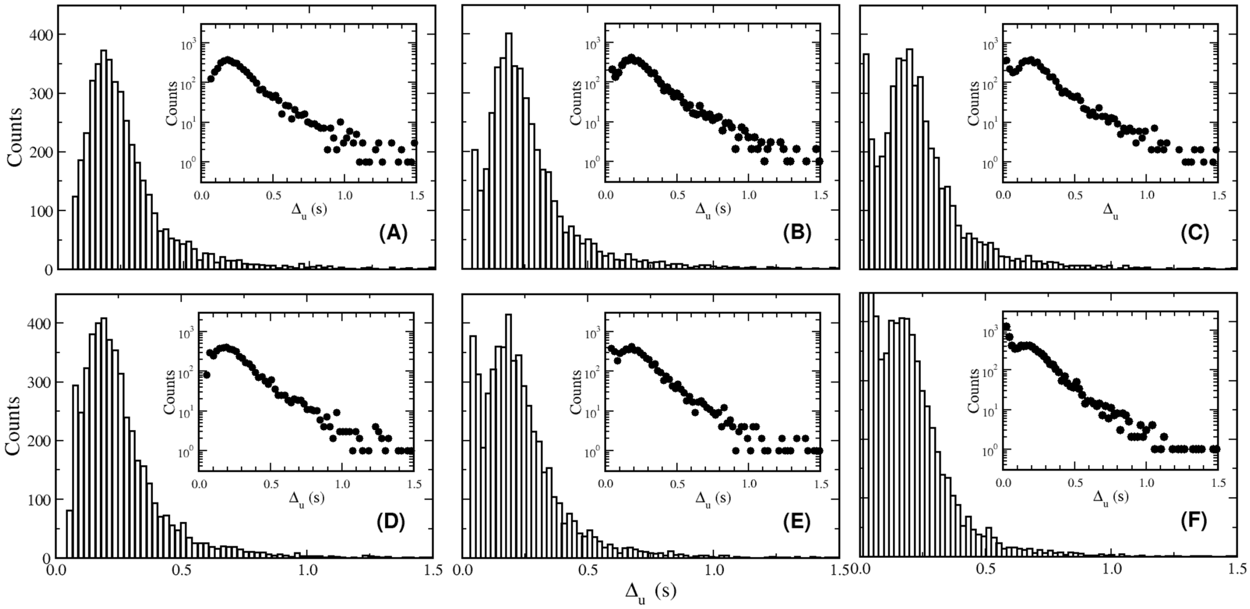

Figure S5: Effects of thresholding and filtering on dwell-time Δ_u histogram. For each histogram, Δ_u were determined as described in the text using different values of c and k. The combinations of three low-pass filters, $c = 30$ (left column), 20 (middle column), and 10 ms (right column), and two thresholds, $k = 75\%$ (top panels (A-C)) and 50% (bottom panels (D-F)), were tested. It is seen that more short intervals, or incomplete switching events, contribute to the histograms as c or k decreases. Nevertheless, even when $c = 10\,\mathrm{ms} < \bar{t}_s$, the histograms still decay non-monotonically.

Viscoelastic Responses Times of Cell Body to Motor Reversals

Most motile bacteria use rotating helices, the flagella, to propel themselves in fluids. Although the physical principle of propulsion appears to be universal, its implementation varies considerably among different bacteria. One observes that some bacteria are peritrichously flagellated, such as *E. coli*, *B. subtilis* and *S. typhimurium*; some are monotrichously flagellated, such as *V. alginolyticus*, *C. crescentus*, and *P. haloplanktis*; some are lophotrichously flagellated, such as *V. fischeri* and *Geobacter*; and still others having their flagella embedded in the cell body, such as spirochaetes (*Treponema*, *Borrelia*, *Leptospira*). In the latter case, the whole cell body undulates as the flagella rotates inside, shedding circular waves from one end of body to the other.

While it is not known why and how these different flagellation patterns arise, it is certain that they are the remarkable results of evolution by natural selection. The selective force appears to work its way to minute details, such as the size and the shape of a hook and a flagellum (filament), and their mechanical properties. Take for example the commonly studied bacteria *E. coli* and *V. alginolyticus.* Because of their different flagellation patterns, there is a fundamental difference in the manner by which force and torque are transmitted to the cell body in these bacteria. For *E. coli*, multiple flagella are connected to motors at their bases via elastic hooks that *bend* by 90 degrees [12]. The hook as well as the flagella are relatively soft compared to that of *V. alginolyticus* [13, 14], allowing multiple flagella to coalesce into a coherent bundle during runs and to disperse during tumbles. Due to multiple motors and flagella involved, bundle formation and dissociation involve complicated dynamics, making it difficult to study motor fluctuations using free-swimming cells. Indeed, casual observations of *E. coli* swimming show that run-to-tumble and tumble-to-run transitions are not very sharp. To study motor fluctuations in *E. coli*, scientists monitor rotations of a small bead that is tethered to a flagellar hook in the absence of the filament [7, 15].

In contrast, the marine bacterium *V. alginolyticus* has a polar flagellum that is connected to the motor at its base by a *straight* hook [12]. In this simple body layout, the flagellum and cell body are aligned so that the force and the torque are transmitted along the cell-body axis. Aided by relatively large flexural rigidities EI of the hook and the filament, our calculation below shows that the response time of the cell body to flagellar motor speed fluctuations is very short, $< 10^{-4}$ s, consistent with our observations and others [16].

The accuracy of measuring dwell-time distributions, $P(\Delta_f)$ and $P(\Delta_b)$, depends on how well the interval times Δ_f and Δ_b can be determined. This in turn depends on how precisely one can determine the moment when a flagellar motor changes its direction. Here two limiting issues need to be considered: (i) the limit set by the measurements, and (ii) the limit set by the intrinsic response time of the cell body to a change in the motor speed. As (i) has already been addressed in the main text, here only (ii) will be analyzed. Treating the flagellar hook and the filament as elastic elements, one can calculate how a local strain induced by the motor propagates along the hook-filament complex and finally causes the cell body to react. We denote the response time by the hook, the filament, and the cell body by τ_h, τ_f, and τ_b, respectively, and the total response time is a sum of them.

(a) Estimate τ_f: Based on the dark-field microscopic measurement by Nishitoba et al. [17], Takano and his coworkers analyzed small deformations of *V. alginolyticus*' flagellum

using the linear elastic theory of Kirchhoff [18]. By comparing the numerical result with the measurement, they estimated the flexural stiffness of flagellar filament to be $EI \sim 10 - 15\,\text{pN} \cdot \mu\text{m}^2$. During the forward or backward swimming, the viscous force per length along the azimuthal θ direction is $f_\theta \approx 2$ pN/μm [18]. Nishitoba et al.'s measurement also showed that when the helix flagellum is pushing or pulling the cell body, the helix tightens or loosens slightly giving rise to a change in the number of turns by ~0.08 in both directions [18]. This allows us to estimate the torsional stiffness GJ of the filament. For the flagellar filament, the angular displacement θ and the force f_θ are related by the Hooke's law,

$$f_\theta L_f R_f = -GJ\frac{\theta - \theta_0}{L_f},$$

where R_f is the radius of the helix, L_f is the contour length of the flagellum, and $\theta - \theta_0 = 0.08 \times 2\pi$ is the twist angle. Using flagellar geometric parameters of *V. alginolyticus* [18], $R_f = 0.23\,\mu$m and $L_f = 5.5\,\mu$m, we found the torsional stiffness $GJ = 28$ pN$\cdot\mu$m^2. For an elastic coil embedded in a viscous fluid, a local twist θ will relax, and the equation of motion of θ is determined by balancing the elastic torques with the viscous ones. This yields

$$C_t R_f \sqrt{R_f^2 + (\frac{\lambda}{2\pi})^2} \partial_t \theta = GJ\frac{\partial^2\theta}{\partial s^2},$$

where C_t is the tangential dragging coefficient per unit length, λ is the pitch, and s is the distance along the contour of the helix. Thus a local twist at one end of the flagellum transmitted to the other end diffusively with a time scale $\tau_f = L^2 C_t R\sqrt{R^2 + (\frac{\lambda}{2\pi})^2}/GJ$. Using the measured $\lambda = 1.27\,\mu$m and the calculated drag coefficient $C_t = \frac{4\pi\eta}{\ln(2q/r_f)+1/2}$, where $\eta = 0.01$ cP is the viscosity of water, $r_f = 16$ nm is the radius of the filament [19, 18], and $q = 0.09\Lambda$ with $\Lambda = 1.57\,\mu$m being the pitch along the contour of the flagellum [20], we found $\tau_f \simeq 3 \times 10^{-4}$ s. Likewise, one can also estimate the compressional relaxation time of the helical coil, yielding nearly the same result. We note that this estimation is consistent with the numerical simulation of Vogel and Stark [21].

(b) Estimate τ_h: Repeat the above linear elastic theory for the hook, the characteristic time is found to be $\propto 4\pi r_h^2 L_h^2 \eta/GJ$, where the radius and length of the hook are $r_h \simeq 0.01\,\mu$m and $L_h \simeq 0.08\,\mu$m, respectively [12]. Although there is no direct measurement, if we assume that the hook is homogeneous and isotropic, it can be estimated that for the hook, $GJ = EI/(1+\nu) \simeq 2.7 \times 10^{-2}\,\text{pN} \cdot \mu\text{m}^2$, where $EI = 3.6 \times 10^{-2}$ pN$\cdot\mu$m^2 and the Poisson ratio $\nu = 1/3$ [16]. The elastic relaxation time estimated in this way, 3×10^{-7} s, is very small and cannot be relevant to our experiment.

On the other hand, experiments using *E. coli* cells reveal that the torsional spring constant of the flagellar hook exhibits strong nonlinearity [13]. Using an optical tweezers to wind and unwind a hook that is connected to a locked flagellar motor, Block et al. discovered that the torsional spring constant is ~0.4 pN μm /rad up to about $\phi_c \simeq 100^o$ of twist, and it then becomes more than an order of magnitude stiffer. In other words, for *E. coli*, once the motor twists the hook over ϕ_c the hook can be considered rigid. Since the length of the flagellar hook of *V. alginolyticus* is $\sim$ 80 nm while that of *E. coli* is $\sim$ 50 nm [22, 23, 12] for *V. alginolyticus,* ϕ_c is expected to be correspondingly greater, or about half turn (if the rigidity of the hooks of *E. coli* and *V. alginolyticus* are the same). *V. alginolyticus* uses a

sodium motor to power its flagellum, and in the steady state, the motor rotates at an angular frequency of $\sim$ 600 Hz when the motility buffer contains 30 mM NaCl [24, 25]. However when motor reverses its direction, the rotation speed is not constant but increases in an almost linear fashion [16]. Thus, the average speed may be taken as $\bar{f} \sim 300$ Hz. This yields a rough estimate of $\tau_h (\equiv \phi_c/\bar{f}) \simeq 0.5/300\,\mathrm{Hz} \simeq 2$ ms. This is a conservative estimate in the sense that while a twist density is introduced at the base of the hook by the flagellar motor, it is released at the distal end. As a result τ_h is expected to be somewhat longer. A recent experiment using fast video imaging shows that upon a motor reversal from CW to CCW rotation, or a transition from backward to forward swimming, the cell body backtracks for $\sim 10\,\mathrm{ms}$ before it is deflected to a new direction. Interestingly the backtracking can be resolved frame by frame at an interval of 1 ms, suggesting that even during this unwinding period the displacement of the cell-body follows closely the rotation of the motor [16]. The sudden change in the swimming direction, which we termed a flick [26], was interpreted as a buckling instability when the rigidity of the hook is at its lowest and can be associated with the loading time of the hook which is about $10 - 20$ ms.

(c) Estimate τ_b: Here we approximate the cell as an ellipsoid with a semi-major axis $a \simeq 1.5\,\mu$m and a semi-minor axis $b \simeq 0.5\,\mu$m. The translational diffusion coefficient D_1 along the cell-body semi-major axis is given by [27],

$$D_1 = \frac{k_B T}{4\pi\eta a}(\ln\frac{2a}{b} - \frac{1}{2}) \tag{1}$$

where k_B is the Boltzmann constant and T is the temperature. This gives $D_1 \simeq 2.8 \times 10^{-13}\,\mathrm{m}^2/\mathrm{s}$ at room temperature. Balancing the inertial force with the viscous force yields the momentum relaxation time $\tau_b = \frac{4}{3}\pi a b^2 \rho D_1/k_B T \approx 10^{-7}$ s, where $\rho \simeq 1\,\mathrm{g/cm}^3$ is the mass density of the cell. This time is far too short to be relevant to our experiment.

The above back-of-the-envelope calculations show that the duration of a flick, or the time it takes for the cell body to reorient, $\tau_r \sim 10{-}20\,\mathrm{ms} \gg \tau_f$, τ_b, τ_h, is the longest relaxation time for the cell body reacting to CW$\to$CCW motor reversals. Although translational motions of the cell body can still be resolved for time less than τ_r by high-speed video imaging [16], this time scale is relevant for our experiment since at the normal video speed, cell reorientation is a major signature of CW$\to$CCW transitions seen under the microscope (see Fig. 6 (C, D) and Ref. [26]).

A swimming interval, Δ_f or Δ_b, consists of two motor reversals, and therefore the minimal interval length must be $\Delta_{min} \simeq \tau_r$ or 20 ms, which is comparable to our video resolution $\pm 16.7\,\mathrm{ms}$. We note that the measured $P(\Delta_f)$ and $P(\Delta_b)$ are peaked at ~ 270 and ~ 370 ms, respectively, which are an order of magnitude greater than Δ_{min}, and these PDFs drop to nearly zero in the neighborhood of Δ_{min} as seen in Fig. 3. Physically, the elastic hook behaves like a low-pass filter that "masks" those short intervals ($\Delta_s < \Delta_{min}$) when the cell body is unable to respond and "let goes" those long intervals ($\Delta_s > \Delta_{min}$) when the cell body is able to respond, where $s \in \{f, b\}$. The fact that the probability of observing small Δ_s drops precipitously suggests that inhibition of these short intervals is intrinsic to the flagellar motor switch of *V. alginolyticus*.

Uncertainties in Determining Dwell Times Δ_f and Δ_b

V. alginolyticus swims at an average speed of 55 μm/s. Therefore during one video frame, $\Delta t = 33$ ms, the average displacement of the cell along its axis is $\sim 1.8\,\mu$m, which is about 7.5 pixels in our setup. This is significantly greater than the displacement by diffusion in the same interval, $\delta_{diff} = \sqrt{2D_1\Delta t} = 0.14\,\mu$m, which is much less than one pixel in our video images. Likewise we can compute the smearing effect due to rotational diffusion of the cell body. The rotational diffusion coefficient D_2 is given by [27]:

$$D_2 = \frac{3k_BT}{8\pi\eta a^3}(\ln\frac{2a}{b} - \frac{1}{2}) \simeq 0.19\,\mathrm{rad}^2/\mathrm{s}. \tag{2}$$

The typical rotation of the cell body due to diffusion over $\Delta t = 33$ ms is $\delta_{rdiff} = \sqrt{2D_2\Delta t} = 0.11$ rad.

Based on the above calculation, we set up an objective criterion for determining the moment of a motor reversal and the associated uncertainty: During a motor reversal, if the displacement of a cell is less than 0.5 μm ($\sim 3\delta_{diff}$) and the cell body's orientation changes less than 0.33 rad ($\sim 3\delta_{rdiff}$) between two consecutive frames, the motor state during the second frame is considered unknown and the uncertainty in deciding the transition moment increases by ± 16.7 ms. The uncertainties σ_f and σ_b for each Δ_f and Δ_b can thus be obtained, and the result is presented in Fig. S6. The figure shows that majority of motor reversal events occur rapidly with σ_f and σ_b being less than 66 ms. The frequency of observing large uncertainties drops by more than an order of magnitude for σ_f, $\sigma_b > 66$ ms. This justifies the resolution limit, the shaded areas in Fig. 3, presented in the main text.

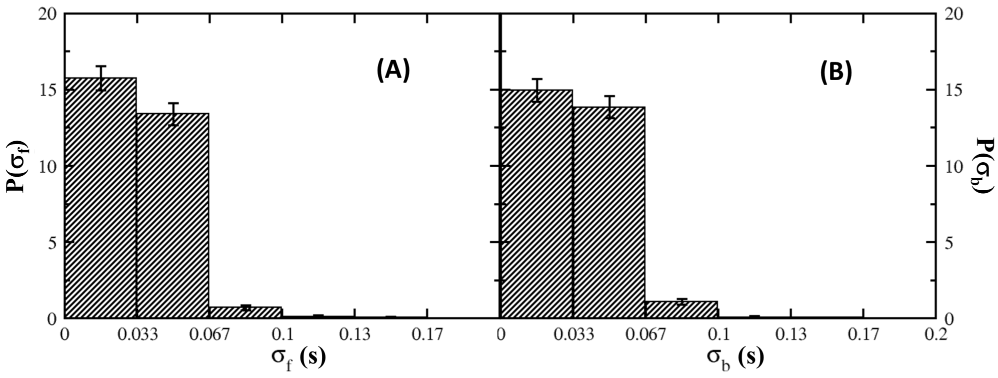

Figure S6: PDFs of σ_f and σ_b. The plots depict the distribution of errors in determining the forward dwell time Δ_f (A) and the backward dwell time Δ_b (B). As can be seen, majority of the intervals can be determined with an uncertainty less than 66 ms. The frequency of uncertainties greater than 66 ms is very small.

References

[1] Kawagishi I, Maekawa Y, Atsumi T, Homma M, Imae Y. Isolation of the polar and lateral flagellum-defective mutants in Vibrio alginolyticus and identification of their flagellar driving energy sources. J Bacteriol. 1995;177:5158–5160.

[2] Tokuda H, Nakamura T, Unemoto T. Potassium ion is required for the generation of pH-dependent membrane potential and ΔpH by the marine bacterium Vibrio alginolyticus. Biochemistry. 1981;20:4198–4203.

[3] Kojima S, Asai Y, Atsumi T, Kawagishi I, Homma M. Na+-driven flagellar motor resistant to phenamil, an amiloride analog, caused by mutations in putative channel components1. Journal of Molecular Biology. 1999;285(4):1537 – 1547.

[4] Magariyama Y, Ichiba M, Nakata K, Baba K, Ohtani T, Kudo S. Difference in bacterial motion between forward and backward swimming caused by the wall effect. Biophys J. 2005;88:3648–3658.

[5] Berke AP, Turner L, Berg HC, Lauga E. Hydrodynamic attraction of swimming microorganisms by surfaces. Phys Rev Lett. 2008;101:038102.

[6] Fahrner KA, Ryu WR, Berg HC. Bacterial flagellar switching under load. Nature. 1965;423.

[7] Yuan JH, Fahrner KA, Berg HC. Switching of the bacterial flagellar motor near zero load. J Mol Biol. 2009;390:390–400.

[8] Altindal T, Chattopadhyay S, Wu XL. Bacterial Chemotaxis in an Optical Trap. PLoS ONE. 2011;6:e18231.

[9] Chattopadhyay S, Moldovan R, Yeung C, Wu XL. Swimming efficiency of bacterium Escherichiacoli. Proceedings of the National Academy of Sciences. 2006;103(37):13712–13717.

[10] Min TL, Mears PJ, Chubiz LM, Rao CV, Golding I, Chemla YR. High-resolution, long-term characterization of bacterial motility using optical tweezers. Nat Meth. 2009;6(11):831–835.

[11] Mears PJ, Koirala S, Rao CV, Golding I, Chemla YR. Escherichia coli swimming is robust against variations in flagellar number. eLife. 2014;3:e01916.

[12] Terashima H, Fukuoka H, Yakushi T, Kojima S, Homma M. The Vibrio motor proteins, MotX and MotY, are associated with the basal body of Na+-driven flagella and required for stator formation. Mol Microbiol. 2006;62:1170–1180.

[13] Block SM, Blair DF, Berg HC. Compliance of bacterial flagella measured with optical tweezers. Nature. 1989;338(6215):514–518. 10.1038/338514a0.

[14] Sen A, Nandy RK, Ghosh AN. Elasticity of flagellar hooks. Journal of Electron Microscopy. 2004;53(3):305–309.

[15] Bai F, Branch RW, Nicolau DV, Pilizota T, Steel BC, Maini PK, et al. Conformational spread as a mechanism for cooperativity in the bacterial flagellar switch. Science. 2010;327:685–689.

[16] Son K, Guasto JS, Stocker R. Bacteria can exploit a flagellar buckling instability to change direction. Nat Phys. 2013;9(8):494–498.

[17] Nishitoba M, Imai N, Magariyama Y, Kudo S. Observation of bacterial flagellar deformation with laser dark-field microscope. 47th Spring Meeting of Japan Soc Appl Phys and Related Soc (in Japanese). 2000;p. 30a–D–5.

[18] Takano Y, Yoshida K, Kudo S, Nishitoba M, Magariyama Y. Analysis of small deformation of helical flagellum of swimming Vibrio alginolyticus. JSME Int J Ser C. 2003;46:1241–1247.

[19] Magariyama Y, Sugiyama S, Muramoto K, Kawagishi I, Imae Y, Kudo S. Simultaneous measurement of bacterial flagellar rotation rate and swimming speed. Biophys J. 1995;69:2154–2162.

[20] Lighthill J. Flagellar Hydrodynamics. SIAM Review. 1976;18(2):161–230.

[21] Vogel R, Stark H. Force-extension curves of bacterial flagella. The European Physical Journal E. 2010;33(3):259–271.

[22] Hirano T, Yamaguchi S, Oosawa K, Aizawa S. Roles of FliK and FlhB in determination of flagellar hook length in Salmonella typhimurium. Journal of Bacteriology. 1994;176(17):5439–5449.

[23] Shibata S, Takahashi N, Chevance FFV, Karlinsey JE, Hughes KT, Aizawa SI. FliK regulates flagellar hook length as an internal ruler. Molecular Microbiology. 2007;64(5):1404–1415.

[24] Sowa Y, Hotta H, Homma M, Ishijima A. Torque-speed relationship of the Na+-driven flagellar motor of Vibrio alginolyticus. J Mol Biol. 2003;327:1043–1051.

[25] Chattopadhyay S, Wu XL. The effect of long-range hydrodynamic interaction on the swimming of a single bacterium. Biophysical Journal. 2009;96:2023–2028.

[26] Xie L, Altindal T, Chattopadhyay S, Wu XL. Bacterial flagellum as a propeller and as a rudder for efficient chemotaxis. Proc Natl Acad Sci USA. 2011;108:2246–2251.

[27] Berg HC. Random Walks in Biology. New Jersey: Princeton University Press; 1993.
